# Seeing and deciding to help: neural and attentional mechanisms of prosociality in younger and older adults

**DOI:** 10.1093/scan/nsag078

**Published:** 2026-09-24

**Authors:** Duo Li, Yuan Cao, Bryant P H Hui, Ping Li, Hui Zhang, Jian Ou, David H K Shum

**Affiliations:** Department of Rehabilitation Sciences, The Hong Kong Polytechnic University, Hong Kong, China; Department of Social Work and Social Administration, The University of Hong Kong, Hong Kong, China; Department of Applied Social Sciences, The Hong Kong Polytechnic University, Hong Kong, China; Department of Language Science and Technology, The Hong Kong Polytechnic University, Hong Kong, China; Department of Rehabilitation Sciences, The Hong Kong Polytechnic University, Hong Kong, China; Department of Rehabilitation Sciences, The Hong Kong Polytechnic University, Hong Kong, China; Department of Rehabilitation Sciences, The Hong Kong Polytechnic University, Hong Kong, China; Research Institute for Smart Ageing, The Hong Kong Polytechnic University, Hong Kong, China

**Keywords:** prosociality, altruism, functional magnetic resonance imaging, eye tracking, psychophysiological interaction

## Abstract

Prosociality plays a crucial role in social functioning across the lifespan. However, the neural and attentional mechanisms underlying age-related differences in prosociality remain to be understudied. In this study, we used task-based functional magnetic resonance imaging and eye tracking to investigate the behavioural, neural, and attentional mechanisms underlying prosocial intentions in 35 younger adults aged 18–31 and 35 older adults aged 60–72 across helping, sharing, and comforting. Behaviourally, older adults reported a greater likelihood of helping than younger adults, while no age differences were observed in sharing or comforting. Older adults showed reduced activation in regions implicated in mentalising and cognitive control, including the right temporoparietal junction and right dorsolateral prefrontal cortex, during sharing and comforting. Older adults also showed attenuated connectivity between prefrontal and temporoparietal regions during sharing. Eye-tracking results revealed that older adults were more likely than younger adults to visually scan recipients across conditions. Brain–behaviour analyses indicated that helping intentions were associated with stronger connectivity of cognitive control regions with mentalising regions and weaker connectivity with affective processing regions. These findings suggest that distinct neural activation, functional connectivity, and attentional deployment patterns support age-related differences in prosociality, highlighting the value of a multimodal approach to understanding prosocial decision-making throughout adulthood.

## Introduction

According to the World Giving Report (Charities Aid Foundation 2025), in 2024, 64% of people worldwide donated money to charitable causes, and 26% volunteered their time to help others. Such voluntary behaviours that benefit others are referred to as prosociality (Eisenberg and Miller 1987). Dunfield’s (2014) comprehensive framework classifies interpersonal prosocial behaviours as helping, sharing, and comforting, which correspond to instrumental, material, and emotional needs, respectively. Neuroimaging research has revealed that prosociality engages mentalising and empathy-related brain networks (Luo 2018; Bellucci *et al*. 2020). However, despite increasing interest in the study of prosocial tendencies across the lifespan, how ageing influences aspects of the neural system that support prosocial decision-making remains unclear.

### Age differences in prosociality

Behavioural evidence suggests that individuals’ prosocial motivation may be enhanced later in life. Compared with younger adults, older adults often report higher levels of empathic concern, donate more to charity, and engage more frequently in volunteering (Sze *et al*. 2012, Beadle and de la Vega 2019, Nakamura *et al*. 2025). These age-related differences have been interpreted from the perspective of socioemotional selectivity theory (Carstensen *et al*. 1999), which proposes that older individuals are more likely to prioritise emotionally meaningful experiences than their younger counterparts. However, the effects of age appear to vary across the different forms of prosociality (Mayr and Freund 2020). A recent meta-analysis found that older adults were more prosocial than younger adults in terms of sharing but not in terms of helping or comforting (Li *et al*. 2024), although research on comforting behaviour among adults remains very limited.

### Neural mechanisms underlying prosociality

Neuroimaging research has shown that mentalising regions, such as the temporoparietal junction (TPJ) and ventromedial prefrontal cortex (vmPFC), are typically recruited during prosocial behaviours (Moll *et al*. 2006). For example, Park *et al*. (2017) asked two groups of younger adults (*n *= 24 per group) to spend money either on themselves or on others. Subsequent functional magnetic resonance imaging (fMRI) studies showed greater TPJ activation in those who had spent money on others than in those who had spent money on themselves, suggesting enhanced engagement of mentalising processes during prosocial behaviour. In another study (Coll *et al*. 2017), prosociality was operationalized as the time spent by participants in alleviating others’ pain while viewing images of patients with shoulder pain. Viewing painful (vs non-painful) images elicited stronger activation in the middle orbital gyrus and middle temporal gyrus regions, which are thought to support mentalising and subsequent helping. A meta-analysis of 67 fMRI studies revealed consistent involvement of the dorsolateral prefrontal cortex (dlPFC), middle cingulate cortex (MCC), posterior cingulate cortex (PCC), and vmPFC (Bellucci *et al*. 2020). However, most of the studies included in this meta-analysis involved younger (*n *= 65) and middle-aged adults (*n *= 2), none included older adults. In summary, several cortical regions have been implicated in prosocial processes, although the precise pattern of activation has varied across experimental paradigms.

In addition to the activation of individual cortical regions, prosociality relies on functional connectivity between regions. For instance, stronger connectivity between the right TPJ and vmPFC was associated with more generous sharing decisions among 27 younger adults (Strombach *et al*. 2015), while greater connectivity between the right superior temporal gyrus and PCC predicted more prosocial economic choices among 30 younger adults (Sun *et al*. 2018). In the context of helping, connectivity between the medial prefrontal cortex (mPFC) and striatum was linked to prosocial actions during a virtual ball-toss task in a sample of 21 younger adults (Kawamichi *et al*. 2019). However, no research to date has examined connectivity associated with comforting behaviour or intentions.

### Neural mechanisms underlying age differences in prosociality

According to the general benevolence model (Hubbard *et al*. 2016), increased prosociality in later life may reflect age-related changes in the way the brain represents the subjective value of outcomes for oneself and others. In this context, “neural utility” refers to valuation-related neural responses that track the motivational or rewarding significance of prosocial outcomes. Using a variant of the dictator game in which participants decided whether to endorse the deduction of charitable donations from their own accounts, greater activation of the vmPFC, nucleus accumbens, and perigenual anterior cingulate cortex (ACC) was found to be associated with a more prosocial disposition and with ageing. Because these regions have been widely implicated in reward processing, subjective valuation, and affective–motivational evaluation, the authors interpreted this pattern as evidence that prosocial outcomes may carry greater subjective value in later life. In another relevant study involving the ultimatum game, in which participants were asked to choose whether to accept monetary offers from a virtual partner, older adults exhibited greater dlPFC activation during decision-making than younger adults (Harlé *et al*. 2012), suggesting that ageing may also alter the recruitment of cognitive control processes during decisions involving fairness or social exchange. Together, these findings suggest that ageing may influence prosocial decision-making through valuation- and control-related neural processes supporting prosocial behaviour.

### Attentional mechanisms underlying prosociality

Researchers have increasingly combined fMRI with eye-tracking to investigate the attentional processes underlying prosocial behaviour. This approach is important because prosocial decisions often begin with the visual detection and interpretation of another person’s need. Before deciding whether to offer help, individuals need to attend to socially relevant cues, such as facial expressions, body posture, and contextual information, and integrate this information with motivational and social–cognitive processes. Eye tracking, therefore, provides an objective index of visual attention to recipients, complementing behavioural ratings that indicate prosocial intentions but not how much individuals visually sample socially relevant cues during the task. Evidence from economic decision-making paradigms suggests that in younger adults, prosocial tendencies are associated with distinct visual attention patterns: specifically, individuals who behave more prosocially exhibit a greater fixation frequency, longer attention to others’ outcomes, and more informative gaze patterns when making monetary decisions (Fiedler *et al*. 2013, Peshkovskaya and Myagkov 2020, Fischbacher *et al*. 2022). However, eye-tracking research has largely operationalized prosociality in terms of monetary sharing, while devoting limited attention to helping or comforting contexts. In particular, age differences in prosocial behaviour may arise not only from altered neural recruitment during decision-making but also from differences in how older and younger adults visually sample recipients’ needs.

Previous eye-tracking studies also have focused largely on younger adults, despite well-documented changes in visual attention with age. A meta-analysis of eight studies reported that, compared with younger adults, older adults spent more time attending to the mouth and less time attending to the eyes when viewing faces (Grainger and Henry 2020). This finding may reflect structural and functional changes in brain regions that support social perception, including the superior temporal sulcus and amygdala (Itier and Batty 2009). Thus, integrating neuroimaging with eye-tracking represents a crucial step towards understanding how interactions between brain function and visual attention influence prosociality in later life.

Multimodal approaches combining fMRI and eye-tracking have enabled the direct investigation of relationships between neural activity, gaze behaviour, and social cognition (Gu *et al*. 2024). Although this approach has been applied in other domains, such as linking gaze behaviour with functional connectivity (Nij Bijvank *et al*. 2021), only one study has combined eye-tracking and fMRI to investigate prosociality. Weng *et al*. (2018) showed that attention to others’ distress was associated with reduced amygdala responses and could predict subsequent sharing behaviour, thus underscoring the value of integrating neural and attentional measures to elucidate prosocial mechanisms. Accordingly, examining behaviour, brain activation, task-dependent connectivity, and eye movements within the same paradigm allows us to test complementary levels of prosocial decision-making: what people intend to do, which neural systems are recruited, how these systems interact, and how visual attention to recipients may support these processes.

### The current study

This study aimed to clarify how ageing influences the behavioural and neural mechanisms of prosociality across distinct contexts. Although older adults tend to be more prosocial than younger adults, little is known about how these neural processes support behavioural differences or are related to attentional sampling. To address these research gaps, task-based fMRI and eye tracking were integrated to capture the neural and attentional processes underlying prosocial decision-making.

We proposed four hypotheses for this study. First, given previous evidence of an association between ageing and increased prosociality, we hypothesised that older adults would report an equal or greater likelihood of engaging in prosociality at the behavioural level than younger adults. Second, at the neural level, we predicted that older and younger adults would exhibit differences in brain activation and connectivity during prosocial decision-making. This is an exploratory hypothesis because limited prior work has examined age differences across helping, sharing, and comforting. Third, as ageing was found to be associated with broader and more effortful information sampling, we hypothesised that older, as compared with younger, adults would exhibit more saccades during prosocial decision-making. Finally, we hypothesised that individual differences in prosociality would be associated with variability in neural activation and connectivity.

## Method

### Participants

Thirty-five younger adults (aged 18–31 years, Mean = 22.51, standard deviation [SD] = 3.73, 14 males) and 35 older adults (aged 60–72 years, Mean = 65.80, SD = 3.03, 15 males) were recruited through advertisements for study participation, which were posted on campus. This sample size was chosen based on previous research, which indicated that 30 participants per group should provide enough power to detect potential group differences in brain activation (Chen *et al*. 2014, Ziaei *et al*. 2021) and eye movements (Circelli *et al*. 2013). Prior to the MRI experiment, we administered the Chinese version of the Montreal Cognitive Assessment (Nasreddine *et al*. 2005) to older participants and excluded those with a score lower than 26 (Rossetti *et al*. 2011). The older adult sample had a mean MoCA score of 28.51 (SD = 1.36). We also obtained written informed consent from the participants prior to the MRI experiment. Younger and older adults were matched with respect to education level and subjective socioeconomic status (SES). Subjective SES was measured using MacArthor’s ladder (Adler *et al*. 2000), on which participants rated their status on a scale from 1 (the lowest) to 10 (the highest).

### Stimuli

Initially, 20 pictures were selected for each type of prosociality. Twenty younger adults and 20 older adults from Hong Kong were recruited to rate the extent to which the target person in each picture required helping, sharing, or comforting, using a 4-point Likert scale (1 = *do not need at all*, 4 = *very much need*). Pictures receiving ratings of 3 or 4 were classified as indicating a need. Only these pictures were included in the fMRI experiment. Finally, 10 pictures per prosociality type were selected, yielding a total of 30 pictures.

### fMRI task

A block design was applied to the fMRI experiment. There were four blocks: helping, sharing, comforting, and control. The order of the four blocks was counterbalanced across the participants. In the prosociality blocks (i.e. helping, sharing, comforting), participants were required to view a picture and rate their likelihood of engaging in the corresponding prosocial behaviour towards the target person in the picture using a 4-point Likert scale (1 = *definitely will not engage in the behaviour*, 4 = *definitely will engage in the behaviour*). Prosocial intention was measured using participants’ button-box responses to this rating scale in each prosocial trial, with higher scores indicating greater willingness to help, share, or comfort the target person. Each prosociality block included 20 trials, with each picture presented twice. In the control block, participants were required to count the number of people in the picture, a very simple task that enabled us to subtract activation associated with visual perception and processing. The control block also included 20 trials, and the pictures were selected from the helping, sharing, or comforting conditions. In each trial, the picture was presented for 8 s followed by a response page where the participant pressed a button (see Fig. 1 for an illustration). Before entering the fMRI scanner, the participants practised three trials for each condition to ensure that they fully understood the experimental requirements.

**Figure 1:**
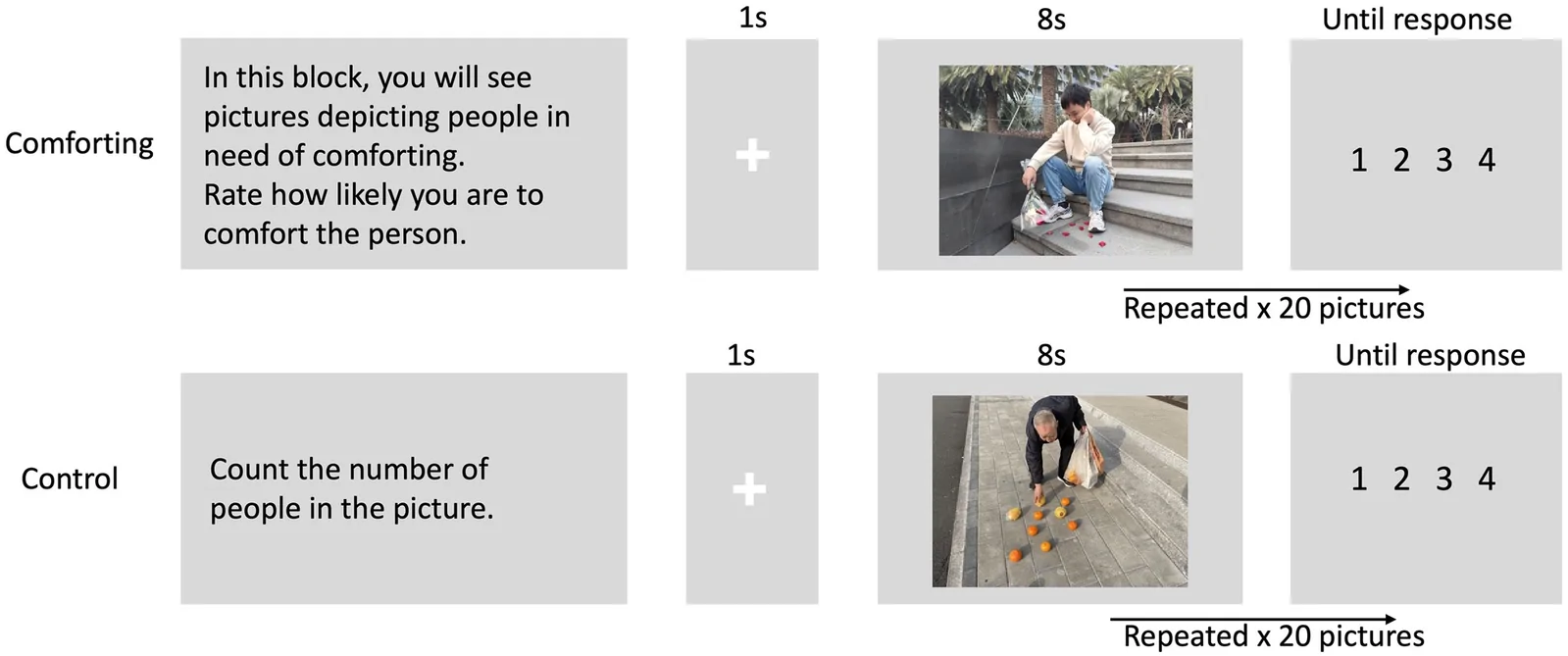
Schematic representation of the fMRI task.

To minimize task-related priming effects and prevent the task from influencing the participants’ self-reports, the Marlowe–Crowne Social Desirability Scale (Crowne and Marlowe 1960, Tao *et al*. 2009) was administered after MRI scanning. The scale was included to account for individual differences in social desirability that might have influenced the participants’ reported prosocial intentions. All participants received 100 HKD as compensation for their time and travel.

### fMRI data acquisition

Functional and structural MRI data were acquired using a 3-T Siemens Magnetom Prisma scanner, the full acquisition parameters are reported in the Supplementary Materials.

### Eye-tracking data acquisition

Participants’ eye movements during the fMRI task were recorded using a long-range mount MRI-compatible eye tracker (EyeLink 1000 Plus, SR Research). The eye-tracker camera was placed at the back of the MRI scanner bore to record the participants’ eye movements using a reflective mirror positioned above the head coil. The distance from the camera to the reflection of the participants’ eyes on the mirror was 120 cm. The recording focused on the participants’ right eye movement. A 9-point calibration and validation protocol was applied to the participants’ eyes, followed by a drift check. Drift checks were repeated before the start of the four blocks.

### Data analysis

#### Behavioural data analysis

The accuracy of the control task was calculated for each participant. Behavioural data from four younger adults and one older adult were excluded due to an accuracy below 80% (Kadlec *et al*. 2024). A linear regression was conducted to control for the influences of social desirability bias and subjective SES on prosociality, and the resulting residuals were used as the adjusted prosocial intentions. Between-group differences in the adjusted prosocial intentions were analysed using the Mann–Whitney *U* test, as the data were not normally distributed.

#### fMRI data analysis

Data from one younger adult and one older adult were excluded due to extreme sleepiness during fMRI scanning. Furthermore, data from one older adult were excluded due to poor image quality. The sample reported in the following fMRI data analysis included 34 younger adults and 33 older adults. Full pre-processing details are provided in the Supplementary Materials.

##### Whole-brain analysis

The fMRI data were preprocessed and analysed using SPM12 in MATLAB. At the individual level, a general linear model analysis was conducted, with four regressors modelling hemodynamic responses at picture onsets of helping, sharing, comforting, and control, and contrast images for each of these four regressors were calculated and averaged across all four fMRI runs. At the group level, these individual-level contrast images were entered into a flexible factorial model with age group as a between-subject factor and condition as a within-subject factor. Three comparisons were tested: (1) main effect of type of prosociality (prosociality minus control), (2) main effect of age group (young minus old, old minus young), and (3) interaction effects of prosociality and age group. Reverse contrasts comparing control with each prosocial condition and overall prosociality were also examined. The threshold for whole-brain results was *P* < .001 (voxelwise, uncorrected), with a cluster-level false discovery rate (FDR) correction at *P* < .05.

##### Psychophysiological interaction

To examine how connectivity between brain regions supports prosociality, we conducted a generalized psychophysiological interaction (gPPI) analysis using the CONN toolbox in MATLAB (Whitfield-Gabrieli and Nieto-Castanon 2012). Eight spherical regions of interest (ROIs, 5-mm radius) were defined a priori based on a recent meta-analysis of fMRI studies on prosociality (Bellucci *et al*. 2020). These ROIs covered the ventromedial prefrontal cortex (vmPFC, MNI: 12, 56, 4), dlPFC (−40, 18, 40), MCC (0, 22, 42), dorsal posterior cingulate cortex (dPCC, 0, −52, 36), TPJ (−54, −70, 11), mPFC (−3, 56, 29), amygdala (20, 2, −12), and supramarginal gyrus (SMG, 60, −32, 32). Multiple comparisons were controlled using the FDR correction across ROI–ROI connections (*P* < .05).

#### Eye-tracking data analysis

Eye-tracking data were successfully collected from 33 younger adults and 22 older adults. Among the participants, failed recording of eye-tracking data was attributed to complicated eye conditions, such as cataract surgery and astigmatism. The eye-tracking data were analysed using the EyeLink Data Viewer (Version: 4.4.1, SR Research). In each picture, an oval area of interest (AOI) covering the face or head of the target person was constructed (Fig. 2). AOI selection was based on previous studies (Hessels *et al*. 2018, Weng *et al*. 2018) suggesting that the facial area of the target person effectively depicts suffering and is vital for understanding attention in social contexts. The saccade count, defined as the total number of saccades into the AOI within the trial, was used to indicate visual attention. Independent sample *t*-tests were conducted to reveal potential differences in saccade by age.

**Figure 2:**
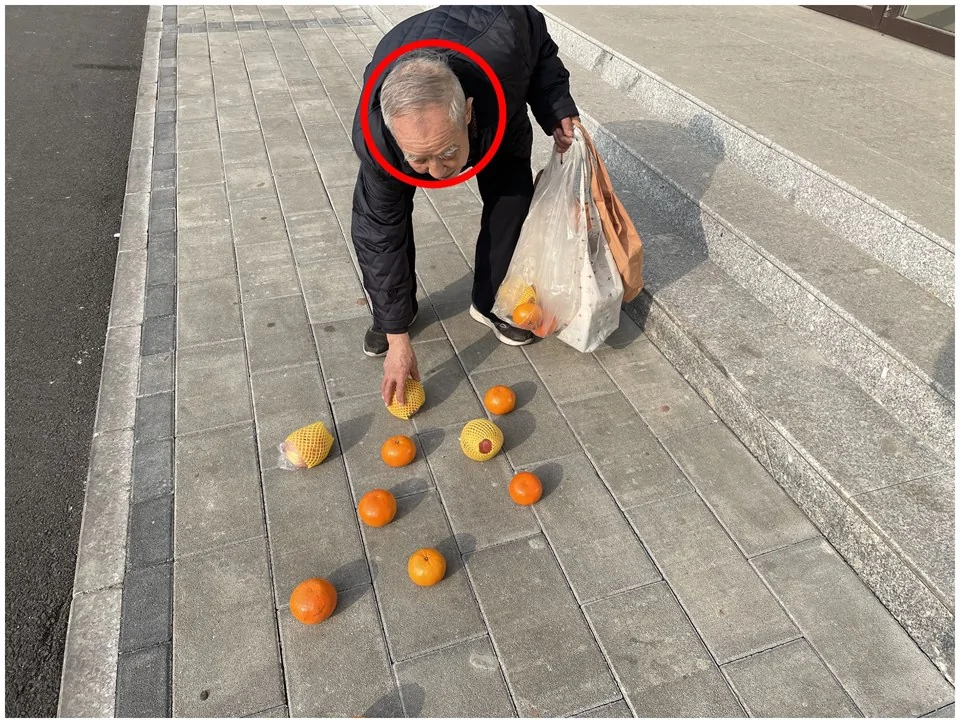
An example of the area of interest in the eye-tracking data analysis.

#### Correlational analyses

To understand why older adults reported higher or lower prosocial intentions in specific domains, we examined the associations of task-related connectivity and the saccade count with self-reported prosociality. These analyses were conducted using partial Pearson correlations after controlling for age group, social desirability, and subjective SES.

Because previous studies have reported links between oculomotor behaviour and large-scale brain connectivity (Dyckman *et al*. 2007, Nij Bijvank *et al*. 2021), we further examined the correlations between the saccade count and PPI connectivity separately for each condition (helping, sharing, comforting) and age group among participants with both valid eye-tracking and fMRI data (younger adults: *n* = 33, older adults: *n* = 22).

## Results

### Sample characteristics

The mean ages of the younger and older adults were 22.30 years (SD = 3.66) and 65.61 years (SD = 2.91), respectively. There were no significant differences in the level of education (*P *= .084) or subjective SES (*P *= .232) between the younger and older adults. Thirty-nine percent of older adults and 57% of younger adults held a certificate/diploma or higher degree. All the participants had completed secondary school. The mean subjective SES was 4.80 (SD = 1.61) among younger adults and 5.09 (SD = 1.31) among older adults, this age-group difference was not statistically significant, *t*(68) = −0.81, *P* = .418.

### Behavioural results

The Mann–Whitney *U* test revealed that older adults (Mean = 3.48, SD = 0.48) were more prosocial than younger adults (Mean = 3.20, SD = 0.42) in terms of helping (*z *= 2.666, *P *= .008) (Fig. 3). However, older and younger adults exhibited a similar likelihood of sharing (Mean = 3.08, SD = 0.75 vs Mean = 2.85, SD = 0.56, *z *= 1.202, *P *= .229) and comforting (Mean = 2.72, SD = 0.59 vs Mean = 2.64, SD = 0.46, *z *= 0.217, *P *= .828). Older adults demonstrated more social desirability (*P *= .002) than younger adults.

**Figure 3:**
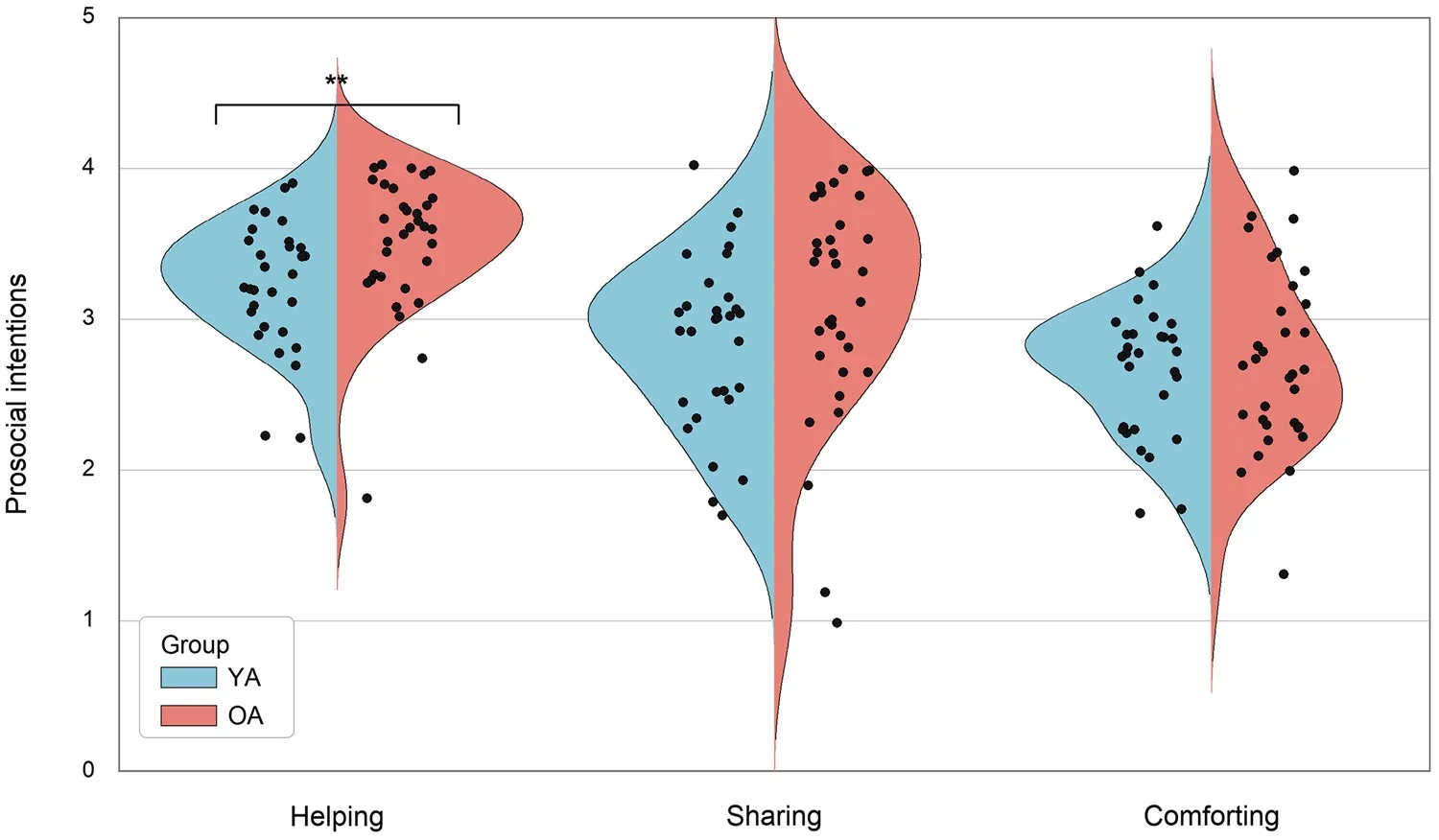
Age differences in self-reported prosocial intentions. **p < .01.

### fMRI results

#### Whole-brain fMRI

Significant main effects of age group, prosociality type, and their interaction were observed with respect to the whole-brain fMRI results. In younger adults, greater activation was detected in the ACC (peak MNI = 3, 53, 20, *k* = 916, *z* = 5.08), right dlPFC (peak MNI = 36, 26, 32, *k* = 34, *z* = 3.92), right TPJ (peak MNI = 54, −58, 11, *k* = 52, *z* = 3.85), and several additional regions under prosocial conditions (collapsed across helping, sharing, and comforting) relative to the control condition (Table 1). In contrast, no significant clusters with respect to prosocial vs control were observed in older adults. The reverse contrasts (control > helping, control > sharing, control > comforting, or control > prosociality) did not reveal any significant clusters in either age group.

**Table 1:** Significant clusters of prosociality.

Peak label	Cluster peak coordinates	Cluster size	Peak z-score
Prosociality-control younger			
Anterior cingulate cortex	3 53 20	916	5.08
Left precentral gyrus	−51 −1 44	30	4.11
Right dorsolateral prefrontal cortex	36 26 32	34	3.92
Right temporoparietal junction	54 −58 11	52	3.85
Right inferior frontal gyrus	30 23 −10	42	3.85
Right cerebellum	42 −61 −31	39	3.74
Right angular gyrus	−48 −64 23	104	3.65
Left inferior parietal lobe	−45 −31 50	42	3.56
Left cerebellum	−36 −67 −25	48	3.50
Posterior cingulate	−3 −58 5	47	3.49
Right inferior frontal gyrus	51 23 −1	23	3.48
Precuneus	−3 −55 32	23	3.31
Prosociality-control older			
None			
Prosociality-control younger > older			
Right superior frontal gyrus	18 44 26	1753	5.15
Right temporoparietal junction	51 −58 14	299	4.79
Left claustrum	−24 20 8	56	4.40
Left postcentral gyrus	−45 −31 50	220	4.10
Left middle frontal gyrus	−51 2 41	148	3.65
Right inferior frontal gyrus	27 23 −4	61	3.64
Extra-nuclear	6 −1 2	22	3.51
Left precuneus	0 −67 17	39	3.51
Thalamus	−9 −7 11	29	3.39
Helping-control younger > older			
None			
Sharing-control younger > older			
Right temporoparietal junction	51 −58 14	184	4.66
Right dorsolateral prefrontal cortex	15 44 26	301	4.52
Comforting-control younger > older			
Right dorsolateral prefrontal cortex	24 41 26	1692	5.27
Right temporoparietal junction	51 −58 11	180	4.28
Left postcentral gyrus	−45 −31 50	400	4.13
Left anterior putamen	−24 20 8	26	4.03
Right insular	33 26 −4	91	3.99
Left precentral gyrus	−21 −7 50	42	3.45
Right postcentral gyrus	63 −10 26	79	3.44
Mid-cingulate cortex	6 −10 38	21	3.34
Precuneus	0 −67 17	24	3.25
Right postcentral gyrus	45 −31 44	29	3.20

Compared with younger adults, older adults showed reduced activation in the right superior frontal gyrus (peak MNI = 18, 44, 26, *k* = 1753, *z* = 5.15), right TPJ (peak MNI = 51, −58, 14, *k* = 299, *z* = 4.79), left postcentral gyrus (peak MNI = −45, −31, 50, *k* = 220, *z* = 4.10), and other regions (Table 1, Fig. 4). When each type of prosociality was examined separately, older and younger adults showed comparable activation during helping, whereas older adults exhibited reduced activation in the right TPJ and right dlPFC during sharing and reduced activation in the right dlPFC, right TPJ, left postcentral gyrus, left anterior putamen, and other regions during comforting. No significant clusters were identified through pairwise comparisons between the three prosocial conditions within either age group.

**Figure 4:**
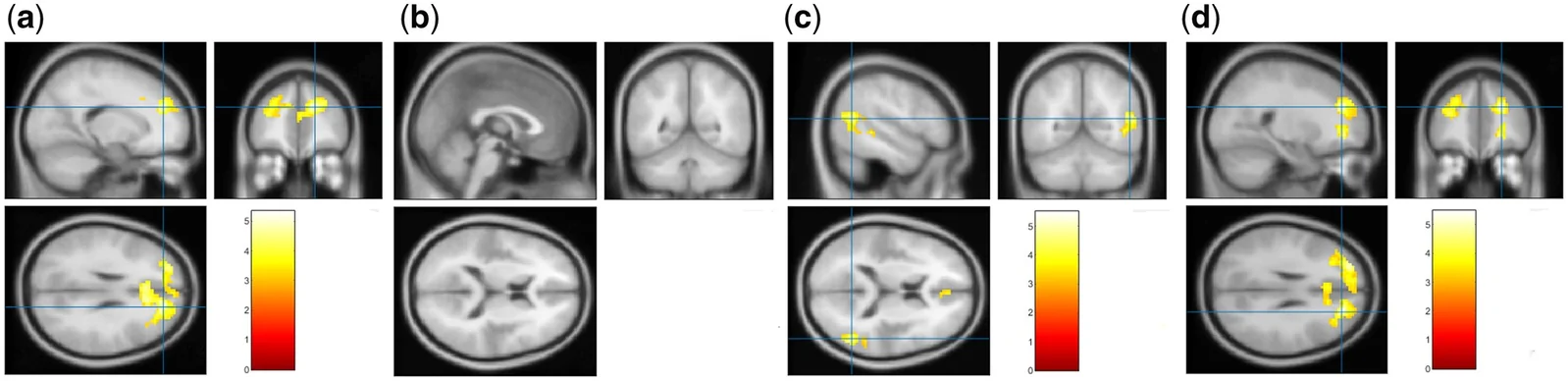
Brain activation maps showing areas of greater activation in younger adults than in older adults. (a) Prosociality vs control. (b) Helping vs control. (c) Sharing vs control. (d) Comforting vs control.

**Figure 5:**
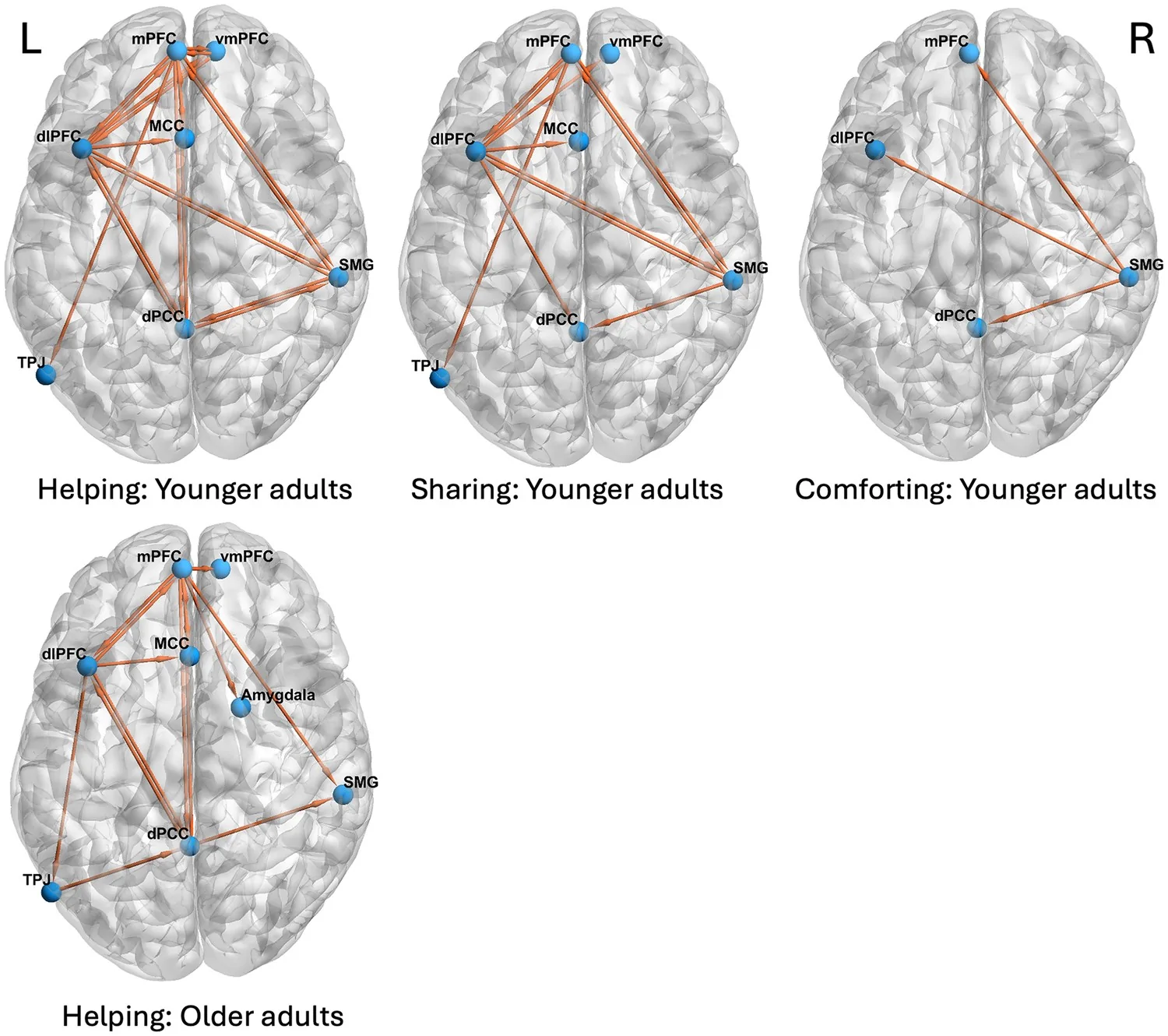
Connectivity patterns associated with prosocial intentions across conditions and age groups. Significant task-related connectivity among regions of interest under the helping, sharing, and comforting conditions in younger adults and older adults. Nodes represent the brain regions included in the analysis, and edges indicate significant connectivity as identified by PPI analyses. Only connectivities that remained after ROI-level multiple comparison correction (FDR-corrected *p* < .05) are displayed. No significant connectivity was observed in older adults under the sharing or comforting condition.

#### Psychophysiological interaction

Both older and younger adults exhibited comparable task-related connectivity under the conditions of helping and comforting. However, older adults demonstrated reduced connectivity between the mPFC and TPJ during sharing relative to younger adults (Fig. 5).

Among younger adults, pairwise comparisons between prosocial conditions did not reveal significant differences in connectivity. In contrast, older adults showed greater dlPFC–TPJ, mPFC–TPJ, mPFC–MCC, and dlPFC–amygdala connectivity during helping than during sharing. Relative to sharing, comforting was associated with greater connectivity between the mPFC–SMG and mPFC–TPJ. No significant differences were found between helping and comforting in older adults.

#### Representational similarity analysis

To further examine whether helping, sharing, and comforting could be distinguished at the multivariate pattern level, we conducted an exploratory ROI-based representational similarity analysis (RSA, see Supplementary Materials). This analysis was performed on unsmoothed functional data to preserve fine-grained multivariate spatial patterns. Pairwise condition RSA showed significant neural pattern separability among helping, sharing, and comforting in both age groups across the ROIs after FDR correction. Cross-minus-within analyses further confirmed that between-condition neural dissimilarity was greater than within-condition dissimilarity. Direct age-group comparisons indicated stronger helping-vs-comforting pattern separation in the mPFC among older adults than younger adults, whereas other age-group differences did not survive correction. Behavioural RSA further suggested that neural representational distances corresponded to stimulus-level differences in prosocial ratings, with stronger neural-behavioural correspondence in older adults in several ROIs, including MCC, vmPFC, and dlPFC.

### Eye-tracking results

Under the helping condition, older adults (Mean = 30.27, SD = 6.98) had more saccades than younger adults (Mean = 22.74, SD = 9.65), *P *= .002. In the sharing condition, older adults (Mean = 29.86, SD = 9.05) also had more saccades than younger adults (Mean = 23.84, SD = 10.18), *P *= .027. Similarly, under the comforting condition, older adults (Mean = 28.38, SD = 7.94) had more saccades than younger adults (Mean = 23.44, SD = 8.60), *P *= .034.

### Correlations among behaviour, brain, and eye-movement measures

Self-reported helping was positively correlated with dPCC–TPJ connectivity (*r *= 0.295, *P* = .023) and dPCC–dlPFC connectivity (*r *= 0.295, *P* = .023), and negatively correlated with dPCC–amygdala connectivity (*r *= −0.430, *P* < .001) and dPCC–SMG connectivity (*r *= −0.271, *P* = .038) after controlling for age group, subjective SES, and social desirability. No significant correlations were observed between the saccade count and self-rated prosociality under any condition.

In younger adults, the saccade count during sharing was negatively correlated with TPJ–amygdala connectivity (*r *= −0.706, *P* < .001). During comforting, the saccade count was negatively correlated with SMG–amygdala connectivity (*r *= −0.526, *P* = .002) and positively correlated with SMG–TPJ connectivity (*r *= 0.524, *P* = .002). No significant correlations were observed during helping. In older adults, no significant correlations were observed.

## Discussion

Using a picture-based paradigm, we found that older adults reported a greater likelihood of helping than younger adults. Neurally, older adults showed reduced activation relative to the control condition compared with younger adults, along with decreased prefrontal–TPJ connectivity. At the attentional level, older adults exhibited greater visual scanning across all prosocial conditions. Brain–behaviour correlations indicated that helping intentions were associated with increased connectivity between mentalising and control regions and reduced connectivity with affective processing regions. Eye–brain correlations in younger adults further suggested interactions between attentional and neural processes. Together, these integrated fMRI and eye-tracking findings provide new evidence on the neural and attentional mechanisms underlying prosociality across age groups.

### Age differences in prosociality at the behavioural level

Older adults reported a greater likelihood of helping others than younger adults, while the two groups reported a similar likelihood of sharing and comforting. This overall pattern of heightened prosociality is consistent with socioemotional selectivity theory, which posits an age-related shift towards emotionally meaningful goals (Carstensen *et al*. 1999). However, the task-specific effects in this study partially diverged from prior findings: whereas previous studies have reported comparable helping across age groups (Gaesser *et al*. 2017, Best and Freund 2021, Li *et al*. 2026) and greater sharing among older adults (Li *et al*. 2024, Pollerhoff *et al*. 2024), we observed an age-related difference only in helping. These discrepancies may reflect methodological factors, including restricted response variability due to the 4-point Likert scale, reduced emotional engagement in the MRI environment, and limited power to detect moderate effects on sharing. Consistent with prior work, we found that comforting did not differ by age (Li *et al*. 2026), although our conclusions should remain tentative given the limited literature on this topic.

### Age differences in prosociality at the neural level

Compared with younger adults, older adults exhibited decreased brain activation during prosocial decision-making, a pattern consistent with previous reviews documenting age-related reductions across a range of cognitive tasks (Reuter-Lorenz and Park 2014, Bernard *et al*. 2020). Importantly, the degree of neural attenuation differed by type of prosociality. No significant age difference in brain activation was observed under the helping condition. Reduced activation was observed in the right TPJ and dlPFC during sharing, while broader reductions, including precentral gyrus, postcentral gyrus, and insular regions, were observed during comforting. The TPJ has been consistently linked to the theory of mind, which is crucial for understanding and responding to others’ needs (Olivo *et al*. 2021). Reduced TPJ activation during sharing may therefore indicate age-related changes in mental state inference when evaluating distributive decisions. Changes in the activation of the precentral and postcentral gyrus, which are associated with sensorimotor processing (Diaz and Yalcinbas 2021, X. Yuan *et al*. 2023), may reflect age-related changes in integrating sensory input (e.g. body gestures of people in the pictures) and motor responses (e.g. physical comforting, such as giving a hug). The broader neural differences observed under the comforting condition relative to the sharing condition suggest that comforting may place greater demands on affective resonance and multimodal integration, and these processes may be particularly sensitive to age-related neural changes (Nashiro *et al*. 2012).

The pattern of reduced TPJ and prefrontal activation observed in older adults may appear inconsistent with a recent study that reported stronger neural responses to others’ pain in older adults than in younger adults (Ferguson *et al*. 2024). This discrepancy may be explained by methodological and conceptual differences. Ferguson *et al*. examined affective empathy for physical and social pain using EEG mu desynchronization, which primarily indexes sensorimotor resonance during pain observation. In contrast, the current study used fMRI to examine prosocial decision-making, where participants evaluated whether they would help, share with, or comfort another person. Thus, our TPJ and prefrontal findings may reflect age-related differences in mentalising, cognitive control, and decision-related integration rather than reduced affective empathy, which is consistent with the possibility that affective resonance and prosocial decision-making rely on partly overlapping but distinct neural mechanisms (Lamm *et al*. 2011, Bellucci *et al*. 2020).

PPI analyses provided further evidence suggesting that ageing alters the coordination of neural systems that support prosociality. Compared with younger adults, older adults exhibited reduced connectivity between the mPFC and TPJ during sharing. Because prefrontal–TPJ connectivity is thought to support the integration of mental state representations with cognitive control and decision-making processes, reduced connectivity may reflect less efficient coordination between perspective-taking and executive systems during sharing decisions. Within older adults, helping was found to elicit stronger connectivity between the TPJ, dlPFC, mPFC, amygdala, and MCC than sharing. This pattern suggests that helping relies on more integrated engagement of affective, motivational, and control networks and may reflect the increased emotional salience or motivational relevance than other types of prosociality. Together, these findings indicate that age differences in prosociality are determined not only by differences in regional activation but also by changes in functional connectivity among key social–cognitive regions.

### Age differences in prosociality at the attentional level

The current study is among the few that have integrated fMRI and eye-tracking to examine the attentional and neural mechanisms underlying prosociality. Compared with younger adults, older adults paid more visual attention to the recipients, as indicated by a greater saccade count, this may reflect broader or less efficient information sampling, consistent with age-related changes in attentional deployment (Barrett *et al*. 2024, L. Yuan *et al*. 2025). Alternatively, older adults may engage in more deliberate and extensive visual scanning as a compensatory strategy to support their social evaluation.

The nonsignificant association between eye movements and self-reported prosocial intentions is consistent with previous findings. Prior studies have found no association between gaze patterns and various aspects of social cognition, such as theory of mind (Grainger *et al*. 2019), emotion recognition (Circelli *et al*. 2013), emotion perception (Grainger and Henry 2020), facial recognition (He *et al*. 2011), or the neural responses evoked by emotional faces (Ziaei *et al*. 2016). Together, these results suggest that eye movements may not provide a direct index of individual differences in social–cognitive outcomes. While eye-tracking is a sensitive measure of visual attention and information sampling, its relationships with higher-level social–cognitive processes may be indirect and context dependent. Further research is needed to clarify how eye movements are related to social cognition across different tasks and populations.

### Age differences in eye–brain–behaviour relationships

Throughout the sample, a greater willingness to help was associated with increased connectivity of the dPCC with the dlPFC and TPJ, as well as decreased connectivity with the amygdala and supramarginal gyrus. This connectivity profile suggests that prosocial motivation may be supported by enhanced integration among the regions involved in valuation and perspective-taking, together with reduced engagement of affective reactivity. These findings suggest that prosocial responses reflect the coordinated recruitment of regulatory and social–cognitive systems, rather than emotional arousal alone.

Beyond brain–behaviour associations, we identified novel relationships between eye movements and task-dependent connectivity. In younger adults, the saccade count during sharing was negatively correlated with connectivity between the TPJ and amygdala, suggesting that increased visual exploration of the recipients may be associated with reduced affective coupling during sharing. This pattern may indicate that younger adults’ processing of others’ needs is more regulated or cognitively mediated when attentional engagement is higher. Similarly, the saccade count was negatively correlated with SMG–amygdala connectivity and positively correlated with SMG–TPJ connectivity, suggesting that increased visual exploration during comforting may be associated with attenuated affective engagement, as well as with enhanced integration within the mentalising network. In contrast, no significant correlations between the saccade count and task-based connectivity were found in older adults. This lack of correlation may partly reflect an overall reduction in connectivity with ageing, as reported in previous studies (La *et al*. 2016). However, this null finding should be interpreted cautiously because the eye–brain analysis in older adults was based on a reduced sample with valid eye-tracking and fMRI data (*n* = 22), which limited statistical power to detect potentially modest associations.

### Neural mechanisms underlying different types of prosociality

Although no significant univariate activation differences were observed between helping, sharing, and comforting, the exploratory RSA suggested that these conditions were distinguishable at the multivariate neural-pattern level. This indicates that different forms of prosociality may recruit overlapping neural systems at the level of overall activation while still differing in their representational patterns. This interpretation is consistent with previous fMRI research proposing that neural substrates are shared between different forms of prosocial behaviour (Cutler and Campbell-Meiklejohn 2019). For example, a meta-analysis of 43 fMRI studies on prosociality used a data-driven approach to categorize prosocial decisions based on brain activation patterns (Rhoads *et al*. 2021). This analysis identified three clusters of prosocial decisions: cooperation, equity, and altruism. Cooperation involved strategic interactions with others (e.g. ultimatum games), equity involved fair or equal resource distribution (e.g. 50–50 splits in dictator games), and altruism involved incurring costs without direct personal gains (e.g. helping or assistance tasks). Importantly, prosocial decisions within the same category exhibited a high degree of inter-relatedness in their neural activation patterns, whereas decisions across categories were more distinct. In the current study, helping, sharing, and comforting could all be classified as altruism as they involve responding to others’ needs without explicit personal gains. Accordingly, these forms of prosociality may involve a common set of brain regions, including the mPFC, ACC, and dlPFC, which are broadly consistent with the regions identified in the current study, while still showing distinguishable multivariate neural patterns. Taken together, these findings suggest that helping, sharing, and comforting may be supported by shared but representationally differentiated neural mechanisms. The exploratory age-group RSA further suggested that older adults showed stronger mPFC pattern separation between helping and comforting, and behavioural RSA indicated stronger neural-behavioural correspondence in several ROIs. However, these findings should be interpreted cautiously because the prosocial conditions were administered in separate blocks; therefore, RSA effects may reflect both condition-related representations and block-related task-set or contextual effects.

### Contributions and implications

The current study provides a more nuanced account of the neural mechanisms underlying different types of prosocial decision-making throughout adulthood. By examining both regional activation and task-dependent connectivity, our study offers new insights into how prosociality is supported by coordinated brain systems. Importantly, our findings help explain older adults’ greater helping intentions by showing that age-related prosociality is linked to behavioural choice and attentional and neural mechanisms. Older adults visually sampled recipients more extensively across prosocial contexts, which may reflect greater attention to others’ needs during decision-making. In addition, helping intentions were associated with stronger connectivity between dPCC, TPJ, and dlPFC, and weaker connectivity with the amygdala. These findings suggest that helping may be supported by the integration of perspective-taking and regulatory processes rather than by affective arousal alone.

These results are important because they show that prosociality in later life should not be treated as a unitary increase in helping others. Instead, age differences appear to vary across helping, sharing, and comforting, and may depend on how older adults attend to recipients’ needs and coordinate social–cognitive neural systems during decision-making. This distinction has practical implications for promoting prosocial engagement in ageing populations: volunteering programmes, charitable campaigns, and intergenerational support initiatives may be more effective when they make others’ needs visually and contextually clear and when they consider the specific type of prosocial behaviour being encouraged. For instance, helping-oriented programmes may benefit from presenting concrete information about recipients’ immediate needs, whereas sharing- or comforting-oriented initiatives may require different forms of contextual or emotional framing. Future applied research is needed to test whether such adaptations can enhance prosocial engagement in real-world settings. Together, our findings underscore the value of integrating neuroimaging and eye-tracking to capture the multifaceted nature of prosociality.

### Limitations and future directions

Several limitations of this research should be acknowledged. First, the social contexts of helping, sharing, and comforting conditions were not fully matched (e.g. comforting typically involves close others, whereas helping often involves strangers), and the participants did not report their perceived relationships to the recipients, these factors may have influenced their behavioural and neural responses. In addition, because helping, sharing, and comforting were presented in separate blocks, the RSA findings cannot fully distinguish condition-specific neural representations from block-related effects such as sustained task set or temporal context. Second, the older adult sample was largely high functioning, which may limit the generalisability of our findings to the broader ageing population. Third, eye-tracking data were available for only a subset of older adults, which reduced the power to detect reliable associations between attentional and neural measures and limited our ability to test whether neural responses mediate the relationship between visual attention and prosocial intentions. Therefore, the present findings should not be interpreted as evidence for a causal pathway from seeing to deciding. Future studies using better-matched stimuli, more representative samples, and larger datasets are needed to clarify age-related differences in prosociality.

## Conclusions

In this study, we applied an integrated fMRI and eye-tracking approach to examine age-related differences in the neural and attentional mechanisms underlying prosocial intentions. Using a picture-based measure of prosocial intention, we found that older adults were more likely to engage in helping than younger adults, and this effect was accompanied by differences in task-dependent brain connectivity. At the neural level, older adults exhibited reduced activation in several brain regions, including the TPJs, prefrontal cortex, and precentral gyrus, suggesting that prosocial decision-making in later life may rely on altered neural recruitment rather than on uniformly increased engagement. By jointly examining the participants’ behavioural responses, neural activation, functional connectivity, and visual attention, the current study provides a more nuanced account of how different types of prosociality are supported throughout adulthood.

## Supplementary Material

nsag078_Supplementary_Data

## Data Availability

The data supporting this study’s findings are available from the first author upon request.
