# Supplementary material for: Seeing and deciding to help: neural and attentional mechanisms of prosociality in younger and older adults
Source: Soc Cogn Affect Neurosci. 2026 Sep 24;21(1):nsag078. doi: 10.1093/scan/nsag078 (PMC13648150; doi:10.1093/scan/nsag078)
Supplement: Supplementary file 1 [file nsag078_supplementary_data.zip › Supplementary_Materials.docx]

fMRI data acquisition

Functional MRI data were recorded using a 3-T scanner (Siemens, Magnetom Prisma) with 64-channel head coil. Four runs of functional images were recorded using a multiband T2* weighted echo-planar sequence (42 interleaved axial slices, TR = 2 s, TE = 27 ms, slice thickness = 3 mm, matrix size = 3 mm × 3 mm, FOV = 192 mm, inter-slice gap = 10% of the slice thickness, flip angle = 80°, acquisition time was approximately 800s depending on the response time of participants, multiband acceleration factor for parallel slice acquisition = 4, coverage omitted the lower extent of the cerebellum). Then, the anatomical T1-weighted MPRAGE image was collected (176 ascending sagittal slices, slice thickness = 1 mm, matrix size = 256 × 256, FOV = 256 mm, TR = 1570 ms, TE = 2.15 ms, acquisition time = 220 s, flip angle = 9°, GRAPPA in-plane acceleration factor = 2). The MRI scanning took around 25 minutes for each participant.

Preprocessing of fMRI data

The fMRI data were preprocessed using the SPM12 toolbox in MATLAB (https://www.fil.ion.ucl.ac.uk/spm/software/spm12/). Images were first spatially realigned to the first image of each MRI run to correct for head motion and slice-timing corrected. Then participants' anatomical images were co-registered to their mean function image created from the realignment step and normalised to the standard Montreal Neurological Institute (MNI) template using segmentation. The derived spatial normalisation parameters were applied to all functional MRI images and finally spatial smoothing was done using a 3D isotropic Gaussian filter of 8 mm full-width/half-maximum. For the RSA, single-trial activation estimates were derived from unsmoothed functional images to preserve fine-grained multivariate spatial patterns.

Supplementary RSA **methods**

To examine whether helping, sharing, and comforting could be distinguished at the multivariate neural-pattern level, we conducted an exploratory ROI-based representational similarity analysis (RSA). Single-trial activation estimates, represented by t-statistic maps derived from least-squares-separate models were extracted from subject-native masks corresponding to the eight a priori prosociality-related ROIs: vmPFC, dlPFC, MCC, dPCC, TPJ, mPFC, amygdala, and SMG. For each participant, ROI, and condition, repeated presentations of the same image were averaged at the stimulus level using image ID alignment. Neural representational dissimilarity matrices (RDMs) were computed as 1 minus the Pearson correlation between stimulus-level activation patterns.

For pairwise condition RSA, binary model RDMs were constructed for helping versus sharing, helping versus comforting, and sharing versus comforting, with within-condition pairs coded as 0 and between-condition pairs coded as 1. Spearman correlations between neural RDMs and model RDMs were calculated for each participant and ROI. To provide a complementary index of condition separation, we also calculated cross-minus-within dissimilarity, defined as the mean between-condition neural dissimilarity minus the mean within-condition neural dissimilarity.

Behavioural RSA was conducted by constructing behavioural RDMs from stimulus-level prosocial intention ratings, defined as the absolute difference in ratings between image pairs. Spearman correlations between neural and behavioural RDMs were computed for all conditions combined and for each pairwise condition comparison. Correlation coefficients were Fisher z transformed before group-level one-sample tests. Benjamini-Hochberg false discovery rate (FDR) correction was applied separately across the 48 pairwise condition RSA tests, 48 cross-minus-within tests, 64 behavioural RSA tests, and 48 pairwise contrasts among condition-pair RSA effects. Exploratory age-group differences were assessed using two-sided label-permutation tests (10,000 permutations), with separate BH-FDR correction across 24 pairwise condition RSA comparisons, 24 cross-minus-within comparisons, and 32 behavioural RSA comparisons.

Supplementary **RSA results**

Pairwise condition RSA showed robust neural pattern separability among helping, sharing, and comforting. All 48 tests were significant after FDR correction (2 groups × 8 ROIs × 3 condition pairs). Cross-minus-within analyses confirmed this pattern: all 48 tests showed greater between-condition than within-condition neural dissimilarity after FDR correction (Table S1). Sanity checks indicated that the split-half diagnostic was not significant and that run-level pattern means and within-condition correlations did not differ significantly across conditions, suggesting that the RSA effects were unlikely to be driven solely by arbitrary trial splitting or gross run-level differences. However, direct contrasts among the three condition-pair RSA effects did not survive FDR correction, indicating that no specific condition pair showed reliably stronger representational separation than the others (Table S3; Figure S1).

Exploratory age-group comparisons suggested that older adults showed stronger helping-versus-comforting pattern separation in the mPFC than younger adults. This effect was observed both for pairwise model RSA (older: mean z = 0.397; younger: mean z = 0.239; permutation p = .0006, q = .0136) and cross-minus-within dissimilarity (older: mean = 0.233; younger: mean = 0.151; permutation p = .0014, q = .0336). No other age-group differences in pairwise model RSA or cross-minus-within dissimilarity survived correction (Table S4).

Behavioural RSA indicated that neural representational distances corresponded to stimulus-level differences in prosocial intention ratings. Across all conditions, behavioural RSA was significant in all eight ROIs in both younger and older adults. Across pairwise scopes, 50 of 64 behavioural RSA tests survived FDR correction (Table S5). Exploratory age-group comparisons suggested stronger neural-behavioural correspondence in older adults in several ROIs, including MCC and vmPFC for all conditions, and MCC, vmPFC, and dlPFC for helping versus comforting (Table S6). These behavioural RSA findings suggest that older adults’ neural representational structure may be more closely aligned with stimulus-level differences in prosocial ratings, although these analyses do not establish that representational differences mediate age-related behavioural differences.

Figure S1. Pairwise RSA effects in younger and older adults
