# Supplementary material for: Seeing and deciding to help: neural and attentional mechanisms of prosociality in younger and older adults
Source: Soc Cogn Affect Neurosci. 2026 Sep 24;21(1):nsag078. doi: 10.1093/scan/nsag078 (PMC13648150; doi:10.1093/scan/nsag078)
Supplement: Supplementary file 1 [file nsag078_supplementary_data.zip › Supplementary tables.docx]

Table S1. Pairwise condition RSA.

| **Group** | **ROI** | **Pair** | **Mean** | **Std** | **Mean_R** | **TValue** | **PValue** | **QValue_BH_FDR** | **Significant_FDR_0_05** |
| --- | --- | --- | --- | --- | --- | --- | --- | --- | --- |
| **Old** | amyg | Helping_vs_Comforting | 0.3775 | 0.1728 | 0.3517 | 12.3595 | 1.62E-13 | 1.02E-12 | TRUE |
| **Old** | amyg | Helping_vs_Sharing | 0.4001 | 0.2191 | 0.3648 | 10.3325 | 1.46E-11 | 2.59E-11 | TRUE |
| **Old** | amyg | Sharing_vs_Comforting | 0.4185 | 0.1800 | 0.3857 | 13.1533 | 3.18E-14 | 3.82E-13 | TRUE |
| **Old** | dlpfc | Helping_vs_Comforting | 0.4433 | 0.2196 | 0.4004 | 11.4178 | 1.23E-12 | 3.70E-12 | TRUE |
| **Old** | dlpfc | Helping_vs_Sharing | 0.4244 | 0.2192 | 0.3853 | 10.9515 | 3.50E-12 | 8.40E-12 | TRUE |
| **Old** | dlpfc | Sharing_vs_Comforting | 0.4586 | 0.2481 | 0.4084 | 10.4575 | 1.09E-11 | 2.09E-11 | TRUE |
| **Old** | dpcc | Helping_vs_Comforting | 0.4431 | 0.2068 | 0.4025 | 12.1221 | 2.68E-13 | 1.29E-12 | TRUE |
| **Old** | dpcc | Helping_vs_Sharing | 0.4654 | 0.2134 | 0.4201 | 12.3360 | 1.71E-13 | 1.02E-12 | TRUE |
| **Old** | dpcc | Sharing_vs_Comforting | 0.4449 | 0.2253 | 0.4010 | 11.1718 | 2.13E-12 | 5.68E-12 | TRUE |
| **Old** | mcc | Helping_vs_Comforting | 0.4068 | 0.1883 | 0.3745 | 12.2207 | 2.18E-13 | 1.16E-12 | TRUE |
| **Old** | mcc | Helping_vs_Sharing | 0.4390 | 0.1790 | 0.4023 | 13.8733 | 7.70E-15 | 1.85E-13 | TRUE |
| **Old** | mcc | Sharing_vs_Comforting | 0.4399 | 0.2243 | 0.3972 | 11.0942 | 2.54E-12 | 6.41E-12 | TRUE |
| **Old** | mpfc | Helping_vs_Comforting | 0.3966 | 0.2075 | 0.3637 | 10.8138 | 4.79E-12 | 1.09E-11 | TRUE |
| **Old** | mpfc | Helping_vs_Sharing | 0.3548 | 0.2164 | 0.3271 | 9.2755 | 1.87E-10 | 2.25E-10 | TRUE |
| **Old** | mpfc | Sharing_vs_Comforting | 0.3773 | 0.2168 | 0.3459 | 9.8432 | 4.67E-11 | 6.22E-11 | TRUE |
| **Old** | smg | Helping_vs_Comforting | 0.4036 | 0.2217 | 0.3668 | 10.2968 | 1.59E-11 | 2.72E-11 | TRUE |
| **Old** | smg | Helping_vs_Sharing | 0.3689 | 0.2083 | 0.3394 | 10.0191 | 3.06E-11 | 4.45E-11 | TRUE |
| **Old** | smg | Sharing_vs_Comforting | 0.4153 | 0.1903 | 0.3814 | 12.3429 | 1.68E-13 | 1.02E-12 | TRUE |
| **Old** | tpj | Helping_vs_Comforting | 0.3552 | 0.1967 | 0.3295 | 10.2157 | 1.92E-11 | 2.98E-11 | TRUE |
| **Old** | tpj | Helping_vs_Sharing | 0.3177 | 0.2067 | 0.2953 | 8.6919 | 8.15E-10 | 9.10E-10 | TRUE |
| **Old** | tpj | Sharing_vs_Comforting | 0.3014 | 0.2299 | 0.2770 | 7.4161 | 2.37E-08 | 2.42E-08 | TRUE |
| **Old** | vmpfc | Helping_vs_Comforting | 0.4129 | 0.2036 | 0.3779 | 11.4735 | 1.09E-12 | 3.49E-12 | TRUE |
| **Old** | vmpfc | Helping_vs_Sharing | 0.4206 | 0.2042 | 0.3840 | 11.6512 | 7.39E-13 | 2.73E-12 | TRUE |
| **Old** | vmpfc | Sharing_vs_Comforting | 0.4582 | 0.1821 | 0.4174 | 14.2312 | 3.88E-15 | 1.85E-13 | TRUE |
| **Young** | amyg | Helping_vs_Comforting | 0.3625 | 0.1844 | 0.3376 | 10.7662 | 1.20E-11 | 2.22E-11 | TRUE |
| **Young** | amyg | Helping_vs_Sharing | 0.3783 | 0.1812 | 0.3515 | 11.2420 | 6.83E-12 | 1.37E-11 | TRUE |
| **Young** | amyg | Sharing_vs_Comforting | 0.4078 | 0.2177 | 0.3716 | 10.0874 | 7.94E-11 | 1.00E-10 | TRUE |
| **Young** | dlpfc | Helping_vs_Comforting | 0.3781 | 0.1705 | 0.3522 | 12.1478 | 6.73E-13 | 2.69E-12 | TRUE |
| **Young** | dlpfc | Helping_vs_Sharing | 0.4299 | 0.1688 | 0.3964 | 13.9444 | 2.17E-14 | 3.48E-13 | TRUE |
| **Young** | dlpfc | Sharing_vs_Comforting | 0.3803 | 0.2008 | 0.3510 | 10.3707 | 2.87E-11 | 4.31E-11 | TRUE |
| **Young** | dpcc | Helping_vs_Comforting | 0.4494 | 0.2060 | 0.4078 | 11.9508 | 1.00E-12 | 3.43E-12 | TRUE |
| **Young** | dpcc | Helping_vs_Sharing | 0.3377 | 0.1669 | 0.3178 | 11.0851 | 6.06E-12 | 1.30E-11 | TRUE |
| **Young** | dpcc | Sharing_vs_Comforting | 0.3710 | 0.2145 | 0.3406 | 9.4722 | 2.23E-10 | 2.61E-10 | TRUE |
| **Young** | mcc | Helping_vs_Comforting | 0.3870 | 0.1608 | 0.3608 | 13.1788 | 9.00E-14 | 8.64E-13 | TRUE |
| **Young** | mcc | Helping_vs_Sharing | 0.3894 | 0.2219 | 0.3559 | 9.6096 | 1.62E-10 | 1.99E-10 | TRUE |
| **Young** | mcc | Sharing_vs_Comforting | 0.3949 | 0.1848 | 0.3648 | 11.7007 | 1.67E-12 | 4.71E-12 | TRUE |
| **Young** | mpfc | Helping_vs_Comforting | 0.2387 | 0.1300 | 0.2306 | 10.0562 | 5.81E-11 | 7.54E-11 | TRUE |
| **Young** | mpfc | Helping_vs_Sharing | 0.2736 | 0.1826 | 0.2588 | 8.2040 | 4.79E-09 | 5.00E-09 | TRUE |
| **Young** | mpfc | Sharing_vs_Comforting | 0.2758 | 0.2026 | 0.2587 | 7.4573 | 3.22E-08 | 3.22E-08 | TRUE |
| **Young** | smg | Helping_vs_Comforting | 0.4171 | 0.2063 | 0.3810 | 11.0735 | 6.22E-12 | 1.30E-11 | TRUE |
| **Young** | smg | Helping_vs_Sharing | 0.4295 | 0.2299 | 0.3874 | 10.2309 | 3.92E-11 | 5.38E-11 | TRUE |
| **Young** | smg | Sharing_vs_Comforting | 0.4520 | 0.2402 | 0.4044 | 10.3045 | 3.33E-11 | 4.70E-11 | TRUE |
| **Young** | tpj | Helping_vs_Comforting | 0.3655 | 0.2229 | 0.3346 | 8.9798 | 7.15E-10 | 8.17E-10 | TRUE |
| **Young** | tpj | Helping_vs_Sharing | 0.3525 | 0.2337 | 0.3220 | 8.2631 | 4.14E-09 | 4.41E-09 | TRUE |
| **Young** | tpj | Sharing_vs_Comforting | 0.2868 | 0.1887 | 0.2700 | 8.3247 | 3.55E-09 | 3.87E-09 | TRUE |
| **Young** | vmpfc | Helping_vs_Comforting | 0.3862 | 0.1999 | 0.3555 | 10.5810 | 1.80E-11 | 2.98E-11 | TRUE |
| **Young** | vmpfc | Helping_vs_Sharing | 0.4216 | 0.1858 | 0.3872 | 12.4293 | 3.84E-13 | 1.68E-12 | TRUE |
| **Young** | vmpfc | Sharing_vs_Comforting | 0.3633 | 0.1886 | 0.3379 | 10.5520 | 1.92E-11 | 2.98E-11 | TRUE |

Table S2. Cross-minus-within neural dissimilarity results.

| **Group** | **ROI** | **Pair** | **Mean** | **Std** | **TValue** | **PValue** | **QValue_BH_FDR** | **Significant_FDR_0_05** |
| --- | --- | --- | --- | --- | --- | --- | --- | --- |
| **Old** | amyg | Helping_vs_Comforting | 0.1891 | 0.0857 | 12.4835 | 1.25E-13 | 6.01E-13 | TRUE |
| **Old** | amyg | Helping_vs_Sharing | 0.2033 | 0.1083 | 10.6180 | 7.51E-12 | 1.39E-11 | TRUE |
| **Old** | amyg | Sharing_vs_Comforting | 0.2115 | 0.0949 | 12.6123 | 9.59E-14 | 5.39E-13 | TRUE |
| **Old** | dlpfc | Helping_vs_Comforting | 0.2213 | 0.1028 | 12.1712 | 2.42E-13 | 8.92E-13 | TRUE |
| **Old** | dlpfc | Helping_vs_Sharing | 0.2174 | 0.1067 | 11.5279 | 9.68E-13 | 2.32E-12 | TRUE |
| **Old** | dlpfc | Sharing_vs_Comforting | 0.2287 | 0.1189 | 10.8797 | 4.12E-12 | 8.60E-12 | TRUE |
| **Old** | dpcc | Helping_vs_Comforting | 0.2183 | 0.1015 | 12.1722 | 2.41E-13 | 8.92E-13 | TRUE |
| **Old** | dpcc | Helping_vs_Sharing | 0.2253 | 0.0947 | 13.4566 | 1.74E-14 | 1.67E-13 | TRUE |
| **Old** | dpcc | Sharing_vs_Comforting | 0.2175 | 0.1018 | 12.0847 | 2.90E-13 | 9.29E-13 | TRUE |
| **Old** | mcc | Helping_vs_Comforting | 0.1960 | 0.0881 | 12.5871 | 1.01E-13 | 5.39E-13 | TRUE |
| **Old** | mcc | Helping_vs_Sharing | 0.2182 | 0.0808 | 15.2783 | 5.60E-16 | 1.34E-14 | TRUE |
| **Old** | mcc | Sharing_vs_Comforting | 0.2238 | 0.1243 | 10.1838 | 2.07E-11 | 3.11E-11 | TRUE |
| **Old** | mpfc | Helping_vs_Comforting | 0.2327 | 0.1073 | 12.2665 | 1.97E-13 | 8.62E-13 | TRUE |
| **Old** | mpfc | Helping_vs_Sharing | 0.2106 | 0.1260 | 9.4539 | 1.20E-10 | 1.45E-10 | TRUE |
| **Old** | mpfc | Sharing_vs_Comforting | 0.2222 | 0.1238 | 10.1534 | 2.23E-11 | 3.24E-11 | TRUE |
| **Old** | smg | Helping_vs_Comforting | 0.2022 | 0.1129 | 10.1284 | 2.36E-11 | 3.33E-11 | TRUE |
| **Old** | smg | Helping_vs_Sharing | 0.1899 | 0.1030 | 10.4325 | 1.15E-11 | 1.94E-11 | TRUE |
| **Old** | smg | Sharing_vs_Comforting | 0.2080 | 0.0932 | 12.6200 | 9.44E-14 | 5.39E-13 | TRUE |
| **Old** | tpj | Helping_vs_Comforting | 0.2216 | 0.1107 | 11.3255 | 1.51E-12 | 3.46E-12 | TRUE |
| **Old** | tpj | Helping_vs_Sharing | 0.2041 | 0.1259 | 9.1699 | 2.43E-10 | 2.72E-10 | TRUE |
| **Old** | tpj | Sharing_vs_Comforting | 0.1978 | 0.1433 | 7.8071 | 8.25E-09 | 8.43E-09 | TRUE |
| **Old** | vmpfc | Helping_vs_Comforting | 0.2007 | 0.0939 | 12.0886 | 2.88E-13 | 9.29E-13 | TRUE |
| **Old** | vmpfc | Helping_vs_Sharing | 0.2088 | 0.0998 | 11.8410 | 4.89E-13 | 1.31E-12 | TRUE |
| **Old** | vmpfc | Sharing_vs_Comforting | 0.2260 | 0.0770 | 16.6098 | 5.53E-17 | 2.65E-15 | TRUE |
| **Young** | amyg | Helping_vs_Comforting | 0.1769 | 0.0943 | 10.2737 | 3.56E-11 | 4.75E-11 | TRUE |
| **Young** | amyg | Helping_vs_Sharing | 0.1895 | 0.0932 | 10.9479 | 1.26E-11 | 2.01E-11 | TRUE |
| **Young** | amyg | Sharing_vs_Comforting | 0.2020 | 0.1048 | 10.3770 | 4.23E-11 | 5.48E-11 | TRUE |
| **Young** | dlpfc | Helping_vs_Comforting | 0.1867 | 0.0817 | 12.5120 | 3.26E-13 | 9.49E-13 | TRUE |
| **Young** | dlpfc | Helping_vs_Sharing | 0.2115 | 0.0812 | 14.2638 | 1.22E-14 | 1.47E-13 | TRUE |
| **Young** | dlpfc | Sharing_vs_Comforting | 0.1888 | 0.0994 | 10.4051 | 2.66E-11 | 3.65E-11 | TRUE |
| **Young** | dpcc | Helping_vs_Comforting | 0.2258 | 0.1011 | 12.2342 | 5.66E-13 | 1.43E-12 | TRUE |
| **Young** | dpcc | Helping_vs_Sharing | 0.1712 | 0.0858 | 10.9255 | 8.54E-12 | 1.52E-11 | TRUE |
| **Young** | dpcc | Sharing_vs_Comforting | 0.1858 | 0.0950 | 10.7114 | 1.36E-11 | 2.10E-11 | TRUE |
| **Young** | mcc | Helping_vs_Comforting | 0.1943 | 0.0728 | 14.6241 | 6.45E-15 | 1.03E-13 | TRUE |
| **Young** | mcc | Helping_vs_Sharing | 0.1946 | 0.1055 | 10.1016 | 5.24E-11 | 6.63E-11 | TRUE |
| **Young** | mcc | Sharing_vs_Comforting | 0.1932 | 0.0847 | 12.4968 | 3.36E-13 | 9.49E-13 | TRUE |
| **Young** | mpfc | Helping_vs_Comforting | 0.1509 | 0.0838 | 9.8669 | 8.94E-11 | 1.10E-10 | TRUE |
| **Young** | mpfc | Helping_vs_Sharing | 0.1739 | 0.1060 | 8.9878 | 7.01E-10 | 7.65E-10 | TRUE |
| **Young** | mpfc | Sharing_vs_Comforting | 0.1756 | 0.1295 | 7.4261 | 3.49E-08 | 3.49E-08 | TRUE |
| **Young** | smg | Helping_vs_Comforting | 0.2126 | 0.1039 | 11.2070 | 4.68E-12 | 9.36E-12 | TRUE |
| **Young** | smg | Helping_vs_Sharing | 0.2245 | 0.1141 | 10.7791 | 1.17E-11 | 1.94E-11 | TRUE |
| **Young** | smg | Sharing_vs_Comforting | 0.2376 | 0.1348 | 9.6489 | 1.48E-10 | 1.73E-10 | TRUE |
| **Young** | tpj | Helping_vs_Comforting | 0.2274 | 0.1315 | 9.4760 | 2.21E-10 | 2.52E-10 | TRUE |
| **Young** | tpj | Helping_vs_Sharing | 0.2255 | 0.1540 | 8.0233 | 7.55E-09 | 7.88E-09 | TRUE |
| **Young** | tpj | Sharing_vs_Comforting | 0.1791 | 0.1183 | 8.2975 | 3.80E-09 | 4.05E-09 | TRUE |
| **Young** | vmpfc | Helping_vs_Comforting | 0.1885 | 0.0931 | 11.0911 | 5.99E-12 | 1.15E-11 | TRUE |
| **Young** | vmpfc | Helping_vs_Sharing | 0.2030 | 0.0825 | 13.4750 | 5.16E-14 | 4.12E-13 | TRUE |
| **Young** | vmpfc | Sharing_vs_Comforting | 0.1769 | 0.0848 | 11.4301 | 2.93E-12 | 6.39E-12 | TRUE |

Table S3. Pairwise contrasts among condition-pair RSA effects.

| **Group** | **ROI** | **Contrast** | **MeanDiff_Z** | **StdDiff_Z** | **TValue** | **PValue** | **QValue_BH_FDR** | **Significant_FDR_0_05** |
| --- | --- | --- | --- | --- | --- | --- | --- | --- |
| **Old** | amyg | Helping_vs_Comforting - Helping_vs_Sharing | -0.0227 | 0.2091 | -0.6128 | 0.5444 | 0.9508 | FALSE |
| **Old** | amyg | Sharing_vs_Comforting - Helping_vs_Sharing | 0.0184 | 0.2483 | 0.4188 | 0.6782 | 0.9672 | FALSE |
| **Old** | amyg | Helping_vs_Comforting - Sharing_vs_Comforting | -0.0410 | 0.2423 | -0.9580 | 0.3455 | 0.9409 | FALSE |
| **Old** | dlpfc | Helping_vs_Comforting - Helping_vs_Sharing | 0.0189 | 0.2400 | 0.4458 | 0.6589 | 0.9672 | FALSE |
| **Old** | dlpfc | Sharing_vs_Comforting - Helping_vs_Sharing | 0.0342 | 0.2198 | 0.8813 | 0.3850 | 0.9409 | FALSE |
| **Old** | dlpfc | Helping_vs_Comforting - Sharing_vs_Comforting | -0.0153 | 0.2768 | -0.3132 | 0.7562 | 0.9845 | FALSE |
| **Old** | dpcc | Helping_vs_Comforting - Helping_vs_Sharing | -0.0223 | 0.2302 | -0.5481 | 0.5876 | 0.9672 | FALSE |
| **Old** | dpcc | Sharing_vs_Comforting - Helping_vs_Sharing | -0.0205 | 0.1849 | -0.6271 | 0.5352 | 0.9508 | FALSE |
| **Old** | dpcc | Helping_vs_Comforting - Sharing_vs_Comforting | -0.0018 | 0.2336 | -0.0439 | 0.9653 | 0.9845 | FALSE |
| **Old** | mcc | Helping_vs_Comforting - Helping_vs_Sharing | -0.0322 | 0.2111 | -0.8639 | 0.3943 | 0.9409 | FALSE |
| **Old** | mcc | Sharing_vs_Comforting - Helping_vs_Sharing | 0.0009 | 0.2508 | 0.0196 | 0.9845 | 0.9845 | FALSE |
| **Old** | mcc | Helping_vs_Comforting - Sharing_vs_Comforting | -0.0331 | 0.2794 | -0.6703 | 0.5076 | 0.9508 | FALSE |
| **Old** | mpfc | Helping_vs_Comforting - Helping_vs_Sharing | 0.0418 | 0.2208 | 1.0702 | 0.2928 | 0.9409 | FALSE |
| **Old** | mpfc | Sharing_vs_Comforting - Helping_vs_Sharing | 0.0225 | 0.2629 | 0.4830 | 0.6325 | 0.9672 | FALSE |
| **Old** | mpfc | Helping_vs_Comforting - Sharing_vs_Comforting | 0.0193 | 0.2129 | 0.5135 | 0.6112 | 0.9672 | FALSE |
| **Old** | smg | Helping_vs_Comforting - Helping_vs_Sharing | 0.0347 | 0.1892 | 1.0362 | 0.3081 | 0.9409 | FALSE |
| **Old** | smg | Sharing_vs_Comforting - Helping_vs_Sharing | 0.0464 | 0.2608 | 1.0054 | 0.3225 | 0.9409 | FALSE |
| **Old** | smg | Helping_vs_Comforting - Sharing_vs_Comforting | -0.0117 | 0.2274 | -0.2910 | 0.7730 | 0.9845 | FALSE |
| **Old** | tpj | Helping_vs_Comforting - Helping_vs_Sharing | 0.0375 | 0.2355 | 0.9017 | 0.3741 | 0.9409 | FALSE |
| **Old** | tpj | Sharing_vs_Comforting - Helping_vs_Sharing | -0.0163 | 0.2408 | -0.3828 | 0.7045 | 0.9672 | FALSE |
| **Old** | tpj | Helping_vs_Comforting - Sharing_vs_Comforting | 0.0538 | 0.2336 | 1.3042 | 0.2018 | 0.9409 | FALSE |
| **Old** | vmpfc | Helping_vs_Comforting - Helping_vs_Sharing | -0.0077 | 0.2258 | -0.1931 | 0.8481 | 0.9845 | FALSE |
| **Old** | vmpfc | Sharing_vs_Comforting - Helping_vs_Sharing | 0.0376 | 0.2238 | 0.9500 | 0.3495 | 0.9409 | FALSE |
| **Old** | vmpfc | Helping_vs_Comforting - Sharing_vs_Comforting | -0.0453 | 0.2701 | -0.9484 | 0.3503 | 0.9409 | FALSE |
| **Young** | amyg | Helping_vs_Comforting - Helping_vs_Sharing | -0.0023 | 0.1854 | -0.0654 | 0.9483 | 0.9845 | FALSE |
| **Young** | amyg | Sharing_vs_Comforting - Helping_vs_Sharing | 0.0295 | 0.2403 | 0.6605 | 0.5143 | 0.9508 | FALSE |
| **Young** | amyg | Helping_vs_Comforting - Sharing_vs_Comforting | -0.0317 | 0.2353 | -0.7259 | 0.4739 | 0.9508 | FALSE |
| **Young** | dlpfc | Helping_vs_Comforting - Helping_vs_Sharing | -0.0517 | 0.1739 | -1.6286 | 0.1142 | 0.9409 | FALSE |
| **Young** | dlpfc | Sharing_vs_Comforting - Helping_vs_Sharing | -0.0496 | 0.2295 | -1.1837 | 0.2462 | 0.9409 | FALSE |
| **Young** | dlpfc | Helping_vs_Comforting - Sharing_vs_Comforting | -0.0021 | 0.2437 | -0.0479 | 0.9621 | 0.9845 | FALSE |
| **Young** | dpcc | Helping_vs_Comforting - Helping_vs_Sharing | 0.1116 | 0.2463 | 2.4833 | 0.0190 | 0.9143 | FALSE |
| **Young** | dpcc | Sharing_vs_Comforting - Helping_vs_Sharing | 0.0333 | 0.2187 | 0.8330 | 0.4116 | 0.9409 | FALSE |
| **Young** | dpcc | Helping_vs_Comforting - Sharing_vs_Comforting | 0.0784 | 0.2639 | 1.6268 | 0.1146 | 0.9409 | FALSE |
| **Young** | mcc | Helping_vs_Comforting - Helping_vs_Sharing | -0.0024 | 0.2082 | -0.0625 | 0.9506 | 0.9845 | FALSE |
| **Young** | mcc | Sharing_vs_Comforting - Helping_vs_Sharing | 0.0055 | 0.1729 | 0.1736 | 0.8634 | 0.9845 | FALSE |
| **Young** | mcc | Helping_vs_Comforting - Sharing_vs_Comforting | -0.0079 | 0.2023 | -0.2127 | 0.8331 | 0.9845 | FALSE |
| **Young** | mpfc | Helping_vs_Comforting - Helping_vs_Sharing | -0.0348 | 0.1587 | -1.2024 | 0.2389 | 0.9409 | FALSE |
| **Young** | mpfc | Sharing_vs_Comforting - Helping_vs_Sharing | 0.0022 | 0.2320 | 0.0526 | 0.9584 | 0.9845 | FALSE |
| **Young** | mpfc | Helping_vs_Comforting - Sharing_vs_Comforting | -0.0371 | 0.2323 | -0.8739 | 0.3893 | 0.9409 | FALSE |
| **Young** | smg | Helping_vs_Comforting - Helping_vs_Sharing | -0.0124 | 0.3151 | -0.2155 | 0.8309 | 0.9845 | FALSE |
| **Young** | smg | Sharing_vs_Comforting - Helping_vs_Sharing | 0.0225 | 0.3222 | 0.3820 | 0.7052 | 0.9672 | FALSE |
| **Young** | smg | Helping_vs_Comforting - Sharing_vs_Comforting | -0.0349 | 0.3195 | -0.5978 | 0.5546 | 0.9508 | FALSE |
| **Young** | tpj | Helping_vs_Comforting - Helping_vs_Sharing | 0.0129 | 0.2797 | 0.2534 | 0.8018 | 0.9845 | FALSE |
| **Young** | tpj | Sharing_vs_Comforting - Helping_vs_Sharing | -0.0657 | 0.2304 | -1.5627 | 0.1290 | 0.9409 | FALSE |
| **Young** | tpj | Helping_vs_Comforting - Sharing_vs_Comforting | 0.0787 | 0.2510 | 1.7163 | 0.0968 | 0.9409 | FALSE |
| **Young** | vmpfc | Helping_vs_Comforting - Helping_vs_Sharing | -0.0355 | 0.1672 | -1.1617 | 0.2548 | 0.9409 | FALSE |
| **Young** | vmpfc | Sharing_vs_Comforting - Helping_vs_Sharing | -0.0583 | 0.2095 | -1.5255 | 0.1380 | 0.9409 | FALSE |
| **Young** | vmpfc | Helping_vs_Comforting - Sharing_vs_Comforting | 0.0229 | 0.1978 | 0.6334 | 0.5314 | 0.9508 | FALSE |

Table S4. Age-group comparisons of pairwise RSA.

| **ROI** | **Pair** | **Mean_Young** | **Mean_Old** | **OldMinusYoung** | **P_perm** | **Q_BH** |
| --- | --- | --- | --- | --- | --- | --- |
| **mpfc** | Helping_vs_Comforting | 0.2387 | 0.3966 | 0.1579 | 0.0006 | 0.0136 |
| **dpcc** | Helping_vs_Sharing | 0.3377 | 0.4654 | 0.1277 | 0.0103 | 0.1240 |
| **vmpfc** | Sharing_vs_Comforting | 0.3633 | 0.4582 | 0.0949 | 0.0498 | 0.3686 |
| **mpfc** | Sharing_vs_Comforting | 0.2758 | 0.3773 | 0.1015 | 0.0614 | 0.3686 |
| **mpfc** | Helping_vs_Sharing | 0.2736 | 0.3548 | 0.0812 | 0.1181 | 0.5670 |
| **dlpfc** | Sharing_vs_Comforting | 0.3803 | 0.4586 | 0.0783 | 0.1806 | 0.6086 |
| **dpcc** | Sharing_vs_Comforting | 0.3710 | 0.4449 | 0.0739 | 0.1916 | 0.6086 |
| **dlpfc** | Helping_vs_Comforting | 0.3781 | 0.4433 | 0.0652 | 0.2029 | 0.6086 |
| **smg** | Helping_vs_Sharing | 0.4295 | 0.3689 | -0.0606 | 0.2815 | 0.7506 |
| **mcc** | Helping_vs_Sharing | 0.3894 | 0.4390 | 0.0496 | 0.3319 | 0.7965 |
| **mcc** | Sharing_vs_Comforting | 0.3949 | 0.4399 | 0.0450 | 0.3929 | 0.8572 |
| **smg** | Sharing_vs_Comforting | 0.4520 | 0.4153 | -0.0367 | 0.5035 | 0.9537 |
| **tpj** | Helping_vs_Sharing | 0.3525 | 0.3177 | -0.0349 | 0.5388 | 0.9537 |
| **vmpfc** | Helping_vs_Comforting | 0.3862 | 0.4129 | 0.0267 | 0.6071 | 0.9537 |
| **mcc** | Helping_vs_Comforting | 0.3870 | 0.4068 | 0.0198 | 0.6616 | 0.9537 |
| **amyg** | Helping_vs_Sharing | 0.3783 | 0.4001 | 0.0218 | 0.6770 | 0.9537 |
| **amyg** | Helping_vs_Comforting | 0.3625 | 0.3775 | 0.0149 | 0.7471 | 0.9537 |
| **tpj** | Sharing_vs_Comforting | 0.2868 | 0.3014 | 0.0145 | 0.7958 | 0.9537 |
| **smg** | Helping_vs_Comforting | 0.4171 | 0.4036 | -0.0136 | 0.8019 | 0.9537 |
| **amyg** | Sharing_vs_Comforting | 0.4078 | 0.4185 | 0.0107 | 0.8385 | 0.9537 |
| **tpj** | Helping_vs_Comforting | 0.3655 | 0.3552 | -0.0103 | 0.8502 | 0.9537 |
| **dpcc** | Helping_vs_Comforting | 0.4494 | 0.4431 | -0.0063 | 0.9060 | 0.9537 |
| **dlpfc** | Helping_vs_Sharing | 0.4299 | 0.4244 | -0.0055 | 0.9139 | 0.9537 |
| **vmpfc** | Helping_vs_Sharing | 0.4216 | 0.4206 | -0.0010 | 0.9834 | 0.9834 |

Table S5. Behavioural RSA results.

| **Group** | **ROI** | **Scope** | **Mean** | **Std** | **Mean_R** | **TValue** | **PValue** | **QValue_BH_FDR** | **Significant_FDR_0_05** |
| --- | --- | --- | --- | --- | --- | --- | --- | --- | --- |
| **Old** | amyg | AllConditions | 0.0787 | 0.1012 | 0.0776 | 4.3990 | 0.0001 | 0.0007 | TRUE |
| **Old** | amyg | Helping_vs_Comforting | 0.1052 | 0.1365 | 0.1027 | 4.3584 | 0.0001 | 0.0007 | TRUE |
| **Old** | amyg | Helping_vs_Sharing | 0.0659 | 0.1566 | 0.0636 | 2.3436 | 0.0259 | 0.0353 | TRUE |
| **Old** | amyg | Sharing_vs_Comforting | 0.1092 | 0.1091 | 0.1075 | 5.6584 | 0.0000 | 0.0000 | TRUE |
| **Old** | dlpfc | AllConditions | 0.0741 | 0.1006 | 0.0729 | 4.1629 | 0.0002 | 0.0009 | TRUE |
| **Old** | dlpfc | Helping_vs_Comforting | 0.1195 | 0.1560 | 0.1148 | 4.3338 | 0.0001 | 0.0007 | TRUE |
| **Old** | dlpfc | Helping_vs_Sharing | 0.0306 | 0.1470 | 0.0276 | 1.1578 | 0.2561 | 0.2621 | FALSE |
| **Old** | dlpfc | Sharing_vs_Comforting | 0.1052 | 0.1357 | 0.1028 | 4.3873 | 0.0001 | 0.0007 | TRUE |
| **Old** | dpcc | AllConditions | 0.0594 | 0.1043 | 0.0582 | 3.2224 | 0.0030 | 0.0064 | TRUE |
| **Old** | dpcc | Helping_vs_Comforting | 0.1025 | 0.1693 | 0.0981 | 3.4244 | 0.0018 | 0.0042 | TRUE |
| **Old** | dpcc | Helping_vs_Sharing | 0.0638 | 0.1754 | 0.0589 | 2.0262 | 0.0517 | 0.0649 | FALSE |
| **Old** | dpcc | Sharing_vs_Comforting | 0.0785 | 0.1173 | 0.0772 | 3.7856 | 0.0007 | 0.0022 | TRUE |
| **Old** | mcc | AllConditions | 0.0988 | 0.0973 | 0.0974 | 5.7446 | 0.0000 | 0.0000 | TRUE |
| **Old** | mcc | Helping_vs_Comforting | 0.1314 | 0.1319 | 0.1282 | 5.6357 | 0.0000 | 0.0000 | TRUE |
| **Old** | mcc | Helping_vs_Sharing | 0.0743 | 0.1186 | 0.0730 | 3.4889 | 0.0015 | 0.0038 | TRUE |
| **Old** | mcc | Sharing_vs_Comforting | 0.1095 | 0.1568 | 0.1059 | 3.9494 | 0.0004 | 0.0015 | TRUE |
| **Old** | mpfc | AllConditions | 0.0662 | 0.0865 | 0.0655 | 4.3309 | 0.0001 | 0.0007 | TRUE |
| **Old** | mpfc | Helping_vs_Comforting | 0.1198 | 0.1574 | 0.1155 | 4.3041 | 0.0002 | 0.0007 | TRUE |
| **Old** | mpfc | Helping_vs_Sharing | 0.0469 | 0.0909 | 0.0463 | 2.8716 | 0.0074 | 0.0132 | TRUE |
| **Old** | mpfc | Sharing_vs_Comforting | 0.0685 | 0.1108 | 0.0673 | 3.4968 | 0.0014 | 0.0038 | TRUE |
| **Old** | smg | AllConditions | 0.0636 | 0.0893 | 0.0628 | 4.0262 | 0.0003 | 0.0013 | TRUE |
| **Old** | smg | Helping_vs_Comforting | 0.1226 | 0.1540 | 0.1184 | 4.5041 | 0.0001 | 0.0007 | TRUE |
| **Old** | smg | Helping_vs_Sharing | 0.0274 | 0.1175 | 0.0267 | 1.2997 | 0.2036 | 0.2172 | FALSE |
| **Old** | smg | Sharing_vs_Comforting | 0.0794 | 0.1399 | 0.0772 | 3.2092 | 0.0031 | 0.0064 | TRUE |
| **Old** | tpj | AllConditions | 0.0619 | 0.1053 | 0.0607 | 3.3257 | 0.0023 | 0.0052 | TRUE |
| **Old** | tpj | Helping_vs_Comforting | 0.1137 | 0.1411 | 0.1108 | 4.5588 | 0.0001 | 0.0007 | TRUE |
| **Old** | tpj | Helping_vs_Sharing | 0.0406 | 0.1293 | 0.0393 | 1.7481 | 0.0907 | 0.1053 | FALSE |
| **Old** | tpj | Sharing_vs_Comforting | 0.0687 | 0.1524 | 0.0664 | 2.5509 | 0.0159 | 0.0248 | TRUE |
| **Old** | vmpfc | AllConditions | 0.0845 | 0.0762 | 0.0837 | 6.2743 | 0.0000 | 0.0000 | TRUE |
| **Old** | vmpfc | Helping_vs_Comforting | 0.1506 | 0.1222 | 0.1470 | 6.9690 | 0.0000 | 0.0000 | TRUE |
| **Old** | vmpfc | Helping_vs_Sharing | 0.0496 | 0.1162 | 0.0486 | 2.3779 | 0.0240 | 0.0349 | TRUE |
| **Old** | vmpfc | Sharing_vs_Comforting | 0.1127 | 0.0977 | 0.1112 | 6.5247 | 0.0000 | 0.0000 | TRUE |
| **Young** | amyg | AllConditions | 0.0347 | 0.0576 | 0.0346 | 3.2977 | 0.0026 | 0.0057 | TRUE |
| **Young** | amyg | Helping_vs_Comforting | 0.0303 | 0.0923 | 0.0300 | 1.7975 | 0.0827 | 0.0984 | FALSE |
| **Young** | amyg | Helping_vs_Sharing | 0.0595 | 0.0908 | 0.0589 | 3.5876 | 0.0012 | 0.0035 | TRUE |
| **Young** | amyg | Sharing_vs_Comforting | 0.0450 | 0.1045 | 0.0443 | 2.3616 | 0.0251 | 0.0350 | TRUE |
| **Young** | dlpfc | AllConditions | 0.0290 | 0.0715 | 0.0288 | 2.2249 | 0.0340 | 0.0436 | TRUE |
| **Young** | dlpfc | Helping_vs_Comforting | 0.0229 | 0.1089 | 0.0226 | 1.1538 | 0.2580 | 0.2621 | FALSE |
| **Young** | dlpfc | Helping_vs_Sharing | 0.0366 | 0.1118 | 0.0361 | 1.7955 | 0.0830 | 0.0984 | FALSE |
| **Young** | dlpfc | Sharing_vs_Comforting | 0.0343 | 0.1227 | 0.0333 | 1.5321 | 0.1363 | 0.1479 | FALSE |
| **Young** | dpcc | AllConditions | 0.0405 | 0.0815 | 0.0401 | 2.7233 | 0.0108 | 0.0178 | TRUE |
| **Young** | dpcc | Helping_vs_Comforting | 0.0609 | 0.1062 | 0.0599 | 3.1407 | 0.0039 | 0.0075 | TRUE |
| **Young** | dpcc | Helping_vs_Sharing | 0.0425 | 0.1348 | 0.0416 | 1.7276 | 0.0947 | 0.1063 | FALSE |
| **Young** | dpcc | Sharing_vs_Comforting | 0.0335 | 0.1052 | 0.0328 | 1.7421 | 0.0921 | 0.1053 | FALSE |
| **Young** | mcc | AllConditions | 0.0406 | 0.0725 | 0.0403 | 3.0690 | 0.0046 | 0.0087 | TRUE |
| **Young** | mcc | Helping_vs_Comforting | 0.0301 | 0.0858 | 0.0298 | 1.9192 | 0.0649 | 0.0798 | FALSE |
| **Young** | mcc | Helping_vs_Sharing | 0.0519 | 0.1024 | 0.0511 | 2.7745 | 0.0096 | 0.0161 | TRUE |
| **Young** | mcc | Sharing_vs_Comforting | 0.0369 | 0.1181 | 0.0360 | 1.7099 | 0.0980 | 0.1081 | FALSE |
| **Young** | mpfc | AllConditions | 0.0350 | 0.0742 | 0.0347 | 2.5862 | 0.0150 | 0.0240 | TRUE |
| **Young** | mpfc | Helping_vs_Comforting | 0.0446 | 0.0984 | 0.0441 | 2.4841 | 0.0190 | 0.0290 | TRUE |
| **Young** | mpfc | Helping_vs_Sharing | 0.0287 | 0.1294 | 0.0275 | 1.2139 | 0.2346 | 0.2461 | FALSE |
| **Young** | mpfc | Sharing_vs_Comforting | 0.0470 | 0.1079 | 0.0460 | 2.3836 | 0.0239 | 0.0349 | TRUE |
| **Young** | smg | AllConditions | 0.0453 | 0.0548 | 0.0452 | 4.5300 | 0.0001 | 0.0007 | TRUE |
| **Young** | smg | Helping_vs_Comforting | 0.0555 | 0.0870 | 0.0551 | 3.4951 | 0.0015 | 0.0038 | TRUE |
| **Young** | smg | Helping_vs_Sharing | 0.0683 | 0.1167 | 0.0670 | 3.2067 | 0.0033 | 0.0065 | TRUE |
| **Young** | smg | Sharing_vs_Comforting | 0.0710 | 0.1364 | 0.0690 | 2.8500 | 0.0080 | 0.0138 | TRUE |
| **Young** | tpj | AllConditions | 0.0367 | 0.0561 | 0.0366 | 3.5843 | 0.0012 | 0.0035 | TRUE |
| **Young** | tpj | Helping_vs_Comforting | 0.0682 | 0.1043 | 0.0672 | 3.5825 | 0.0012 | 0.0035 | TRUE |
| **Young** | tpj | Helping_vs_Sharing | 0.0391 | 0.0961 | 0.0387 | 2.2286 | 0.0338 | 0.0436 | TRUE |
| **Young** | tpj | Sharing_vs_Comforting | 0.0103 | 0.1082 | 0.0099 | 0.5204 | 0.6067 | 0.6067 | FALSE |
| **Young** | vmpfc | AllConditions | 0.0352 | 0.0632 | 0.0349 | 3.0467 | 0.0049 | 0.0089 | TRUE |
| **Young** | vmpfc | Helping_vs_Comforting | 0.0621 | 0.0952 | 0.0616 | 3.5742 | 0.0013 | 0.0035 | TRUE |
| **Young** | vmpfc | Helping_vs_Sharing | 0.0350 | 0.0810 | 0.0345 | 2.3675 | 0.0248 | 0.0350 | TRUE |
| **Young** | vmpfc | Sharing_vs_Comforting | 0.0424 | 0.1020 | 0.0416 | 2.2776 | 0.0303 | 0.0404 | TRUE |

Table S6. Age-group comparisons of behavioural RSA.

| **ROI** | **Scope** | **MeanZ_Young** | **MeanZ_Old** | **MeanR_Young** | **MeanR_Old** | **OldMinusYoung_Z** | **P_perm** | **Q_BH** |
| --- | --- | --- | --- | --- | --- | --- | --- | --- |
| **mcc** | Helping_vs_Comforting | 0.0301 | 0.1314 | 0.0301 | 0.1307 | 0.1013 | 0.0004 | 0.0139 |
| **vmpfc** | Helping_vs_Comforting | 0.0621 | 0.1506 | 0.0621 | 0.1494 | 0.0884 | 0.0025 | 0.0405 |
| **dlpfc** | Helping_vs_Comforting | 0.0229 | 0.1195 | 0.0229 | 0.1189 | 0.0966 | 0.0053 | 0.0484 |
| **vmpfc** | AllConditions | 0.0352 | 0.0845 | 0.0351 | 0.0843 | 0.0494 | 0.0075 | 0.0484 |
| **vmpfc** | Sharing_vs_Comforting | 0.0424 | 0.1127 | 0.0424 | 0.1122 | 0.0703 | 0.0079 | 0.0484 |
| **mcc** | AllConditions | 0.0406 | 0.0988 | 0.0406 | 0.0985 | 0.0582 | 0.0091 | 0.0484 |
| **amyg** | Helping_vs_Comforting | 0.0303 | 0.1052 | 0.0303 | 0.1048 | 0.0749 | 0.0146 | 0.0667 |
| **amyg** | Sharing_vs_Comforting | 0.0450 | 0.1092 | 0.0450 | 0.1087 | 0.0641 | 0.0227 | 0.0908 |
| **mpfc** | Helping_vs_Comforting | 0.0446 | 0.1198 | 0.0446 | 0.1192 | 0.0751 | 0.0280 | 0.0996 |
| **dlpfc** | Sharing_vs_Comforting | 0.0343 | 0.1052 | 0.0343 | 0.1049 | 0.0709 | 0.0346 | 0.1060 |
| **smg** | Helping_vs_Comforting | 0.0555 | 0.1226 | 0.0555 | 0.1220 | 0.0671 | 0.0377 | 0.1060 |
| **amyg** | AllConditions | 0.0347 | 0.0787 | 0.0347 | 0.0785 | 0.0440 | 0.0416 | 0.1060 |
| **dlpfc** | AllConditions | 0.0290 | 0.0741 | 0.0290 | 0.0739 | 0.0450 | 0.0452 | 0.1060 |
| **mcc** | Sharing_vs_Comforting | 0.0369 | 0.1095 | 0.0369 | 0.1090 | 0.0726 | 0.0464 | 0.1060 |
| **tpj** | Sharing_vs_Comforting | 0.0103 | 0.0687 | 0.0103 | 0.0686 | 0.0584 | 0.0896 | 0.1911 |
| **dpcc** | Sharing_vs_Comforting | 0.0335 | 0.0785 | 0.0335 | 0.0784 | 0.0450 | 0.1179 | 0.2357 |
| **mpfc** | AllConditions | 0.0350 | 0.0662 | 0.0350 | 0.0661 | 0.0312 | 0.1332 | 0.2507 |
| **tpj** | Helping_vs_Comforting | 0.0682 | 0.1137 | 0.0681 | 0.1132 | 0.0455 | 0.1559 | 0.2772 |
| **smg** | Helping_vs_Sharing | 0.0683 | 0.0274 | 0.0682 | 0.0274 | -0.0409 | 0.1786 | 0.3008 |
| **tpj** | AllConditions | 0.0367 | 0.0619 | 0.0367 | 0.0618 | 0.0252 | 0.2537 | 0.3915 |
| **dpcc** | Helping_vs_Comforting | 0.0609 | 0.1025 | 0.0608 | 0.1021 | 0.0416 | 0.2569 | 0.3915 |
| **smg** | AllConditions | 0.0453 | 0.0636 | 0.0453 | 0.0635 | 0.0182 | 0.3447 | 0.5013 |
| **mcc** | Helping_vs_Sharing | 0.0519 | 0.0743 | 0.0518 | 0.0742 | 0.0224 | 0.4317 | 0.5648 |
| **dpcc** | AllConditions | 0.0405 | 0.0594 | 0.0405 | 0.0593 | 0.0189 | 0.4409 | 0.5648 |
| **mpfc** | Sharing_vs_Comforting | 0.0470 | 0.0685 | 0.0469 | 0.0684 | 0.0216 | 0.4413 | 0.5648 |
| **mpfc** | Helping_vs_Sharing | 0.0287 | 0.0469 | 0.0287 | 0.0468 | 0.0182 | 0.5265 | 0.6480 |
| **vmpfc** | Helping_vs_Sharing | 0.0350 | 0.0496 | 0.0350 | 0.0496 | 0.0146 | 0.5706 | 0.6763 |
| **dpcc** | Helping_vs_Sharing | 0.0425 | 0.0638 | 0.0425 | 0.0637 | 0.0213 | 0.6050 | 0.6915 |
| **smg** | Sharing_vs_Comforting | 0.0710 | 0.0794 | 0.0708 | 0.0792 | 0.0084 | 0.8137 | 0.8922 |
| **amyg** | Helping_vs_Sharing | 0.0595 | 0.0659 | 0.0594 | 0.0658 | 0.0065 | 0.8423 | 0.8922 |
| **dlpfc** | Helping_vs_Sharing | 0.0366 | 0.0306 | 0.0366 | 0.0305 | -0.0061 | 0.8643 | 0.8922 |
| **tpj** | Helping_vs_Sharing | 0.0391 | 0.0406 | 0.0391 | 0.0406 | 0.0015 | 0.9601 | 0.9601 |
